# USP24 induces IL-6 in tumor-associated microenvironment by stabilizing p300 and β-TrCP and promotes cancer malignancy

**DOI:** 10.1038/s41467-018-06178-1

**Published:** 2018-09-28

**Authors:** Yi-Chang Wang, Yu-Syuan Wu, Chia-Yang Hung, Shao-An Wang, Ming-Jer Young, Tsung-I Hsu, Jan-Jong Hung

**Affiliations:** 1 Department of Biotechnology and Bioindustry Sciences, National Cheng Kung University, Tainan, 70101 Taiwan; 2 Institute of Basic Medical Sciences, National Cheng Kung University, Tainan, 70101 Taiwan; 3 The Ph.D. Program for Neural Regenerative Medicine, College of Medical Science and Technology, Taipei Medical University, Taipei, 11031 Taiwan; 4 The Ph.D. Program for Cancer Molecular Biology and Drug Discovery, Taipei Medical University, Taipei, 11031 Taiwan

## Abstract

We have previously demonstrated that USP24 is involved in cancer progression. Here, we found that USP24 expression is upregulated in M2 macrophages and lung cancer cells. Conditioned medium from USP24-knockdown M2 macrophages decreases the migratory and chemotactic activity of lung cancer cells and the angiogenic properties of human microvascular endothelial cell 1 (HMEC-1). *IL-6* expression is significantly decreased in USP24-knockdown M2 macrophages and lung cancer cells, and IL-6-replenished conditioned medium restores the migratory, chemotactic and angiogenetic properties of the cells. USP24 stabilizes p300 and β-TrCP to increase the levels of histone-3 acetylation and NF-κB, and decreases the levels of DNMT1 and IκB, thereby increasing *IL-6* transcription in M2 macrophages and lung cancer cells, results in cancer malignancy finally. IL-6 has previously been a target for cancer drug development. Here, we provide direct evidence to support that USP24 promotes *IL-6* expression, which might be beneficial for cancer therapy.

## Abstract (web summary)

USP24 has previously been reported to be involved in cancer progression. Here, the authors demonstrate that USP24 stabilizes p300 and β-TrCP to increase the levels of NF-κB and histone-3 acetylation, and decrease DNMT1 and IκB levels which promotes *IL-6* expression in M2 macrophages and lung cancer cells.

## Introduction

Deubiquitinases (DUBs) are specific enzymes that regulate multiple cellular functions by modulating ubiquitin molecules. Ubiquitin-specific peptidases belong to the superfamily of DUBs that have been correlated with many human diseases, including cancer progression. More than 50 ubiquitin-specific peptidases have been identified, and most of these enzymes exert their functions by reversing the polyubiquitination or monoubiquitination of target proteins. Malfunction of the ubiquitin system can either enhance the effect of oncogenes or reduce tumor suppressor genes activity, and this system has been implicated in the tumorigenesis of various cancers^1,2^.

Ubiquitin specific peptidase 24 (USP24) is a 2620 amino acid protein that contains one ubiquitin-associated domain (UBA), which binds to the ubiquitin signal on substrate proteins, and one ubiquitin C-terminal hydrolase (UCH) domain, which is the catalytic domain. The function of USP24 is poorly understood, and most studies examining USP24 have focused on the single nucleotide polymorphisms (SNPs) of USP24 that are implicated in Parkinson disease (PD)^3^. In our previous study, we demonstrated that USP24 expression was upregulated in most late-stage lung cancer patients due to increased mRNA stability caused by SNPs or RNA editing^4^. We also found that upregulated USP24 decreases the stability of the methyltransferase Suv39h1 by promoting the expression of mouse double minute 2 homolog (MDM2). Downregulation of Suv39h1 releases downstream genes from inhibition; thereby leading to the expression of metastasis-related genes, such as C-C motif chemokine ligand 5 (*CCL5*) and ADAM metallopeptidase domain 10 (*ADAM10*). Based on these findings, we concluded that upregulated USP24 in cancer cells plays a critical role promoting lung cancer metastasis. In addition to the deregulation of the tumor cell themselves, other cell types in the tumor-associated microenvironment, such as tumor-infiltrating leukocytes (TILs), cancer-associated-fibroblasts, vascular endothelial and stromal cells, can also promote cancer cell malignancy^5,6^. Furthermore, although the tumor-associated microenvironment is extremely important to the metastatic process, the function of USP24 in the tumor-associated microenvironment remains unclear.

Leukocytes found in neoplastic tissues are defined as TILs^7^. TILs are dominated by tumor-associated macrophages, which specifically refers to macrophages found in the tumor-associated microenvironment. The phenotypes of macrophages are the results of microenvironment stimulations and can be classified into M1 and M2 types. Most immune cells located in the tumor-associated microenvironment are M2 macrophages, which are differentiated from peripheral blood monocytes recruited to tumor sites by growth factors and chemokines secreted by the stroma and tumor cells. M2 macrophages express several well-known surface makers, including CD206, and promote cancer cell metastasis by secreting many molecules, including growth factors and other cytokines^8,9^. Interleukin 6 (IL-6) is a cytokine secreted by M2 macrophages that promotes cancer cell metastasis by activating signal transducer and activator of transcription 3 (STAT-3) and repressing E-cadherin expression^10,11^. The regulation of *IL-6* expression in the tumor-associated microenvironment has been studied for many years, and multiple mechanisms have been identified, including genetic and epigenetic regulation.

Previous studies have reported that *IL-6* expression is regulated by several crucial transcription factors, including nuclear factor kappa B (NF-κB) and IκB^12,13^. Therefore, the modification of these transcriptional factors also affects *IL-6* expression and has been used as a therapeutic strategy. Because the ubiquitination and degradation of IκB leads to the liberation and nuclear translocation of NF-κB, leading to *IL-6* transcription, novel therapeutic strategies have been developed to treat multiple myeloma by preventing IκB degradation^14,15^. A previous study also showed that the acetylation of RelA, the p65 subunit of the NF-κB heterodimer, increases NF-κB transcriptional activity by decreasing NF-κB ubiquitination and degradation^16^. In this study, we discovered the underlying mechanisms by which USP24 not only promotes IκB degradation by stabilizing beta-transduction repeat containing E3 ubiquitin protein ligase (β-TrCP) in lung cancer cells but also induces the upregulation of NF-κB in M2 macrophages, resulting in an increase in *IL-6* expression.

In addition to the modification of crucial transcriptional factors, other studies have revealed that epigenetic regulation also modulates *IL-6* expression. The histone acetyltransferase (HAT) p300 is recruited by NF-κB to the *IL-6* promoter and acetylates the core histones, further facilitating *IL-6* transcription^17,18^. An HAT inhibitor was also reported to inhibit *IL-6* expression by preventing the recruitment of p300 to the *IL-6* promoter^19^. Other than histone modification, an inverse correlation between the methylation status of a CpG site in the *IL-6* promoter and the *IL-6* mRNA level was observed in rheumatoid arthritis^20^. The methylation status of specific CpG sites in the *IL-6* promoter is also inversely related to *IL-6*mRNA levels in lung cancer cell lines^21^. In this study, we found that USP24 increased *IL-6* expression by decreasing the stability of DNMT1 by stabilizing β-TrCP in lung cancer cells and increasing the stability of p300 in both lung cancer cells and M2 macrophages, thereby resulting in the acetylation of histone H3 and demethylation of the *IL-6* promoter, respectively.

A detailed and comprehensive understanding of the mechanistic regulation of *IL-6* may provide insight into metastasis prevention. In this study, we found that USP24 is not only upregulated in malignant cancer cells but also in M2 macrophages and participates in cancer metastasis by positively regulating *IL-6* expression through multiple genetic and epigenetic mechanisms. Our finding suggests that USP24 plays a critical role in the regulation of *IL-6* and can serve as a therapeutic target for the prevention of IL-6-mediated metastasis.

## Results

### TILs show high USP24 expression levels

**Fig. 1:**
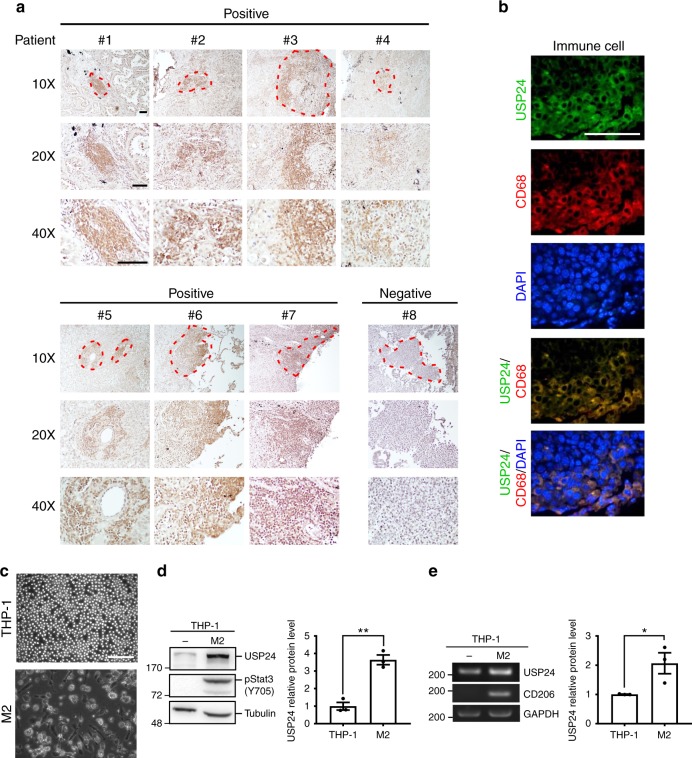
USP24 level in M2 macrophages. **a** Representative images of immunohistochemistry staining of USP24 in lung cancer specimens by using anti-USP24 antibody. Leukocytes were circled with red dashed lines. Scale bar represents 200 μm. **b** Immunofluorescence staining of USP24, CD68, and DAPI in one human lung cancer specimen. Scale bar represents 50 μm. **c** Morphological change of M2 macrophages derived from THP-1 monocytes. Scale bar represents 200 μm. **d**, **e** USP24 protein (*n* = 3) (**d**) and RNA (*n* = 3) (**e**) level in THP-1 and M2 macrophages were analyzed by western blotting and RT-PCR. Results were normalized with tubulin or *GAPDH* level and expressed as fold of control. Data are shown as mean ± SEM, two-tailed unpaired Student’s *t*-test, *P *<* 0.05 and **P *<* 0.01

We previously showed that USP24 is highly expressed in late-stage lung cancer patients and promotes cancer cell metastasis by regulating metastasis-related gene expression^4^. After validating the specificity of anti-USP24 antibodies, USP24 levels were studied in human lung cancer samples using immunohistochemistry (IHC) (Fig. 1a and Supplementary Fig. 6b). We observed several tumor regions with accumulated leukocytes. Leukocytes in neoplastic tissues are termed TILs and were detected by staining for the CD68 marker (Fig. 1b). These TILs expressed higher USP24 levels than the surrounding cancer cells in seven lung cancer patient specimens positive for USP24 expression. We also used immunofluorescence to confirm that the cells highly expressing USP24 were macrophages by staining with the macrophage marker CD68. Because macrophages dominate TILs, and most macrophages in the tumor-associated microenvironment are M2 macrophages. USP24 strongly co-localized with CD68 (Fig. 1b), implying that USP24 might be highly expressed in tumor-associated M2 macrophages and might be involved in tumor-associated macrophage-mediated cancer progression. THP-1 and U937 cells were differentiated into M2 macrophages by upregulating p-STAT3 and *CD206* levels, leading to significantly increased USP24 protein and mRNA levels in M2 macrophages compared with those in THP1 and U937 cells (Fig. 1c–e and Supplementary Fig. 1a).

### USP24 in M2 macrophages increases lung cancer malignancy

**Fig. 2:**
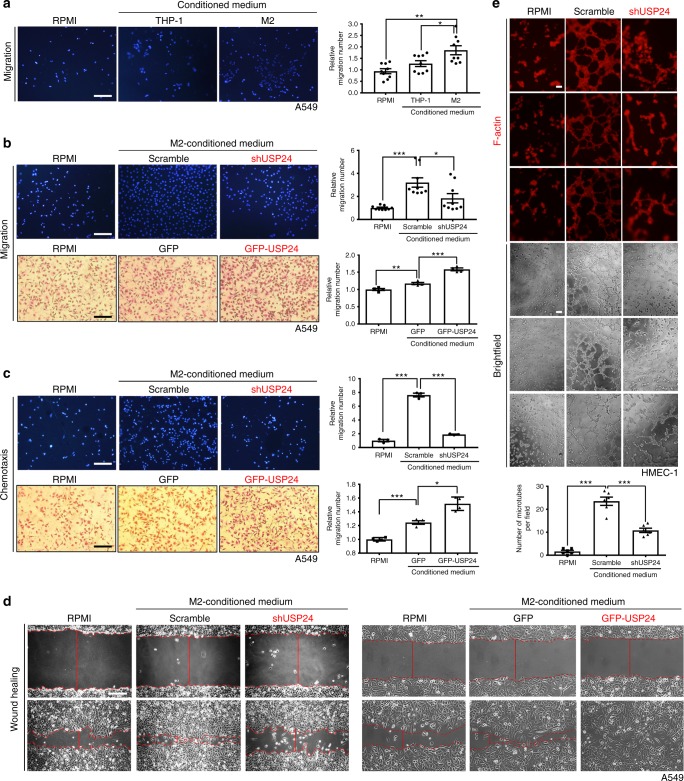
The metastasis-related effects of conditioned medium derived from M2 macrophages. **a** A549 cells were treated with RPMI or conditioned medium derived from THP-1 or M2 macrophages for 24 h, and transwell migration assay was performed to analyze the migratory ability of lung cancer cells (DAPI staining, *n* = 9). Scale bar represents 60 μm. **b**–**d** A549 cells were treated with RPMI or conditioned medium derived from USP24 knockdown or USP24 overexpression M2 macrophages for 24 h, and transwell migration assay (**b**) (DAPI staining, *n* = 9, scale bar: 100 μm; Giemsa staining, *n* = 4, scale bar: 60 μm), chemotactic assay (**c**) (DAPI staining, *n* = 3, scale bar: 100 μm; Giemsa staining, *n* = 4, scale bar: 60 μm) and wound-healing migration assay (**d**) were performed. Wound edges were indicated with red dashed lines and wound width was presented as solid red lines. Scale bar represents 200 μm. **e** HMEC-1 cells were treated with RPMI or conditioned medium derived from scramble- or USP24-knockdown M2 macrophages for 6 h. Cells were stained with F-actin and photographed (*n* = 6). Results were normalized with control and expressed as fold of control. Scale bar represents 200 μm. Data are shown as mean ± SEM, two-tailed unpaired Student’s *t*-test, *P *<* 0.05, **P < 0.01, and ***P < 0.005

Because previous studies have shown that M2 macrophages play important roles in the tumor-associated microenvironment, including tumor promotion, downregulation of adaptive immune responses, and metastasis promotion, by secreting several cytokines and factors, we clarified whether the USP24 upregulation in M2 macrophages affected these cellular functions^22–26^. Initially, we collected conditioned medium from THP-1 and M2 macrophages and treated A549 cells with this conditioned medium (Fig. 2). The conditioned medium derived from M2 macrophages significantly increased the migratory ability of A549 lung cancer cells compared with cells treated with RPMI medium only or THP-1 conditioned medium (Fig. 2a). A549 epithelial lung cancer cells were also treated with conditioned medium collected from M2 macrophages after USP24 knockdown or overexpression (Fig. 2b–d). USP24 knockdown decreased, while USP24 overexpression increased, the transwell chemotactic, and migratory properties of lung cancer cells. In addition to A549 cells, the migration ability of the highly metastatic CL1–5 cell line exhibited downregulated transwell migratory and chemotactic activity after treatment with conditioned medium derived from USP24-knockdown M2 macrophages (Supplementary Fig. 1b, c). Taken together, these data showed that upregulated USP24 in M2 macrophages positively regulates lung cancer malignancy. Previous studies have also reported that M2 macrophages induce angiogenesis^27^. Therefore, we used human microvascular endothelial cell line 1 (HEMC-1) and human umbilical vein endothelial cells (HUVECs) to examine the effect of USP24 on angiogenesis. The tube-formation ability was increased after incubation with M2 macrophage-derived conditioned medium. However, this effect was prevented after USP24 knockdown in the M2 macrophages (Fig. 2e and Supplementary Fig. 1d).

Next, an in vivo animal model was used to clarify the role of USP24 in M2 macrophages-induced metastasis and angiogenesis. CL1–5 epithelial lung cancer cells were mixed with control or USP24-knockdown M2 macrophages and subsequently injected into the backs of SCID mice subcutaneously (Supplementary Fig. 1e). SCID mice were sacrificed two months after injection and the tumor volume and metastatic nodules were examined. The results showed that the volume of the primary tumor formed by CL1–5 was significantly increased when mixed with M2 macrophages. However, the volume of the primary tumor formed by CL1–5 showed no difference when mixed with M2 macrophages after USP24 knockdown. Hepatic metastasis and angiogenesis were also more severe when CL1–5 cells were mixed with M2 macrophages than they were when the cells were mixed with M2 macrophages after USP24 knockdown, suggesting that USP24 expression is essential in M2 macrophages for M2 macrophage-mediated lung cancer malignancy in vivo. In conclusion, USP24 in TAMs is crucial for tumor malignancy, including metastasis and angiogenesis.

### IL-6 upregulation by USP24 increases cancer malignancy

**Fig. 3:**
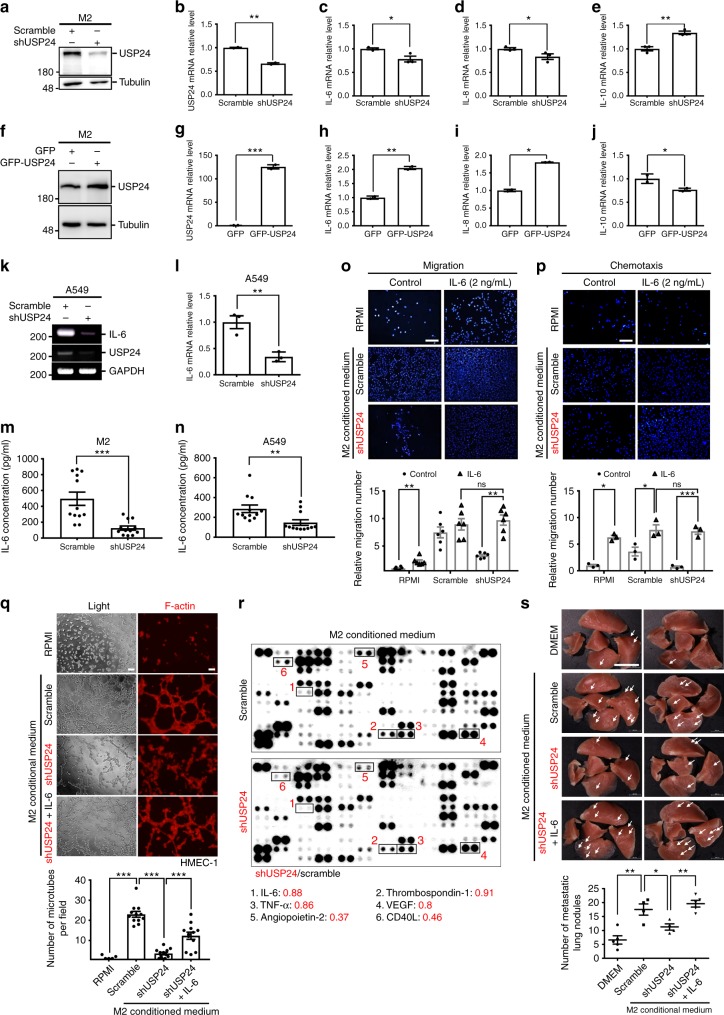
IL-6 regulated by USP24 increases lung cancer metastasis. **a**–**j** Western blot and Q-PCR was utilized to analyze the protein level of USP24 (**a**, **f**) and mRNA levels of *USP24*, *IL-6*, *IL-8*, and *IL-10* in M2 macrophages after USP24 knockdown (**b**–**e**) or overexpression (**g**–**j**) (*n* = 3). **k**, **l** *IL-6* mRNA level in A549 cells was analyzed by RT-PCR (**k**) and Q-PCR (**l**) after USP24 knockdown (*n* = 3). Results of Q-PCR were normalized with *GAPDH* and expressed as fold of control. **m**, **n** IL-6 secretion was analyzed in conditioned medium derived from M2 macrophages (**m**) and A549 cells (**n**) after USP24 knockdown (*n* = 12). **o**–**q** IL-6 was replenished in USP24-knockdown M2 macrophages derived conditioned medium, and migration assay (**o**) (*n* = 6), chemotaxis assay (**p**) (*n* = 3) (DAPI staining, scale bar: 100 μm), and angiogenesis assay (**q**) (*n* = 12) (scale bar: 200 μm.) were analyzed. Results were normalized with control and expressed as fold of control. **r** The levels of IL-6, Thrombospondin-1, TNF-α, VEGF, Angiopoietin-2 and CD40L were studied by using protein array. **s** CL1–5 cells were treated with DMEM (*n* = 5) or conditioned medium derived from scramble knockdown (*n* = 4), USP24 knockdown (*n* = 4) or IL-6 replenished USP24 knockdown (*n* = 5) M2 macrophages for 24 h and injected into SCID mice through tail vein injection. The white arrowheads indicate the metastatic nodules (scale bar: 1 cm). Data are shown as mean ± SEM, two-tailed unpaired Student’s *t*-test, ns for not significant, *P < 0.05, **P < 0.01, and ***P < 0.005

To identify which factor(s) are regulated by USP24 in M2 macrophages during the lung cancer malignancy process, several metastasis-related factors were studied after USP24 knockdown or overexpression in M2 macrophages (Fig. 3a–j and Supplementary Fig. 2a). USP24 positively regulates *IL-6*,* IL-8*, *IL-22*, and *IL-24* expression but negatively regulates *IL-10* and *IL-17* expression. Because IL-6 can activate the NF-κB pathway to express several inflammatory-related genes, including cytokines, USP24-mediated *IL-6* expression might be crucial to control the other cytokines in the tumor-associated microenvironment^28^. Previous studies have shown that IL-6 is secreted not only by the tumor-associated microenvironments but also by the tumor itself^29,30^. Therefore, we assessed the IL-6 levels in the USP24-knockdown A549 cells (Fig. 3k, l). Similar to M2 macrophages, the *IL-6* mRNA level was also dramatically decreased in USP24-knockdown A549 cells. The level of secreted IL-6 in the culture medium was also assessed after USP24 knockdown in M2 macrophages and A549 cells (Fig. 3m, n). The IL-6 levels secreted from M2 macrophages and A549 cells decreased after USP24 knockdown, suggesting that USP24 upregulation in the tumor-associated microenvironment and cancer cells during tumorigenesis increased *IL-6* expression.

To examine whether USP24-mediated IL-6 was indeed functioning in lung cancer metastasis and angiogenesis, IL-6 was added to conditioned medium derived from USP24-knockdown M2 macrophages to address malignant lung cancer cell activity (Fig. 3o–q). IL-6 treatment increased the transwell migratory and chemotactic properties of A549 cells and rescued the migratory, chemotactic and angiogenic activities attenuated by USP24-knockdown conditioned medium. Several angiogenesis-related factors in the conditioned medium were studied by protein array (Fig. 3r). The data indicated that the knockdown of USP24 in M2 macrophages decreased Angiopoirtin-2, CD40L, IL-6, tumor necrosis factor alpha (TNF-α), Thrombospondin-1 and vascular endothelial growth factor (VEGF) levels, indicating that USP24 is involved in angiogenesis. In addition, IL-8 treatment increased cancer malignancy, indicating that not only IL-6 but also other factors regulated by USP24 modulate cancer malignancy (Supplementary Fig. 2b, c). Finally, CL1–5 lung cancer cells pretreated with USP24-knockdown conditioned medium were injected into SCID mice through the tail vein to examine metastatic ability (Fig. 3s). The number of nodules in the lung increased in the group treated with M2 macrophage-conditioned medium, but this effect was lost when USP24-knockdown M2 macrophage conditioned medium was used. After IL-6 was added to the M2 macrophages conditioned medium, this effect was rescued, indicating that the USP24-induced metastasis activity is due to upregulated IL-6, which can be expressed by M2 macrophages or cancer cells.

### USP24 regulates *IL-6* expression by modulating p300 and NF-κB

**Fig. 4:**
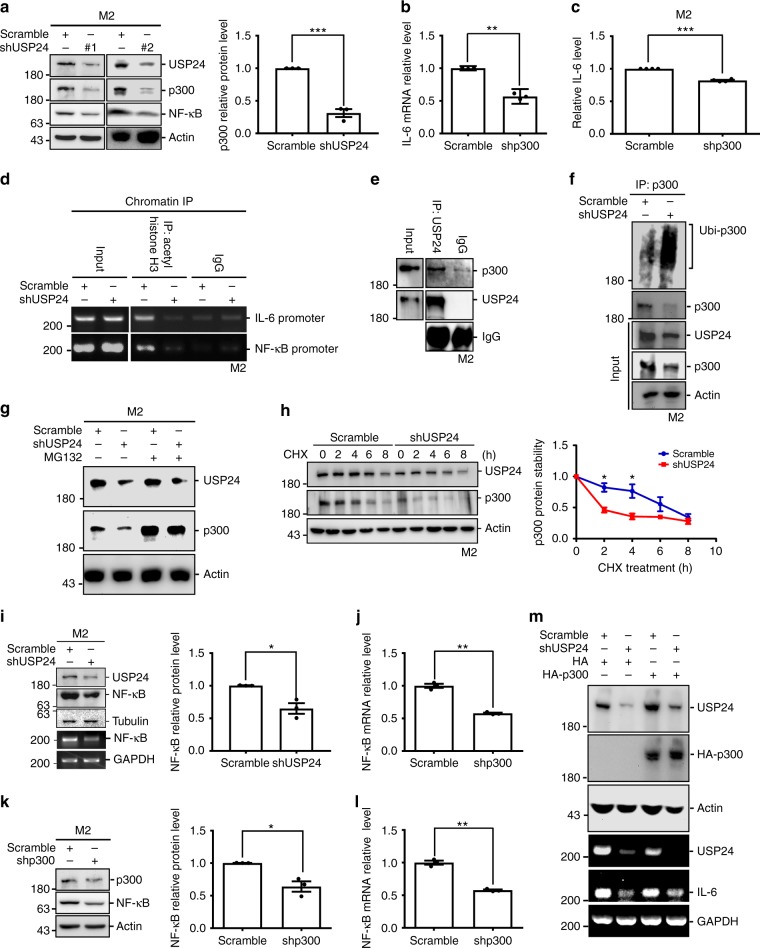
USP24-stabilized p300 increases NF-κB and IL-6 expression in M2 macrophages. **a** Western blotting was used to analyze the indicated protein levels in scramble- and USP24-knockdown M2 macrophages by using two different shRNA clones. **b** Q-PCR was used to analyze *IL-6* RNA level in scramble- and p300-knockdown M2 macrophages. **c** IL-6 level in conditioned medium derived from scramble- and p300-knockdown M2 macrophage was studied by ELISA (*n* = 4). **d** The recruitment of acetyl histone H3 to the promoters of *IL-6* and *NF-κB* in USP24-knockdown M2 macrophages was analyzed by chromatin immunoprecipitation (IP) assay. **e** The lysates were harvested from M2 macrophages for immunoprecipitation with anti-USP24 antibodies. IP samples were used to study the USP24 and p300 levels by western blotting with anti-USP24 and anti-p300 antibodies. **f** The lysates were harvested from scramble- and USP24-knockdown M2 macrophage for immunoprecipitation with anti-p300 antibodies. IP samples were used to study the ubiquitinated p300 by western blotting with anti-ubiquitin antibody. **g** The lysates collected from scramble- or USP24-knockdown M2 macrophages with MG132 treatment were used to study the indicated protein levels by western blotting. **h** Scramble and USP24-knockdown M2 macrophages were treated with CHX and collected at indicated time points. p300 protein stability was analyzed by western blotting. **i**, **j** NF-κB protein and mRNA levels were measured in scramble- and USP24-knockdown M2 macrophages by western blotting, RT-PCR (**i**) and Q-PCR (**j**). **k**, **l** Western blotting (**k**) and Q-PCR (**l**) were used to analyze indicated protein and mRNA levels in scramble- and p300-knockdown M2 macrophages. **m** The lysates were harvested from A549 cells with or without USP24 knockdown or p300 overexpression and analyzed by using the western blotting and RT-PCR. Protein and mRNA levels were quantified after independent experiments (*n* = 3). Results of western blot and Q-PCR were normalized with actin, tubulin or *GAPDH* and expressed as fold of control. Data are shown as mean ± SEM, two-tailed unpaired Student’s *t*-test, *P *<* 0.05, **P < 0 .01, and ***P < 0.005

The detailed mechanisms regarding how USP24 regulates *IL-6* expression in M2 macrophages and lung cancer cells require further clarification. A previous study indicated that p300 and NF-κB regulate IL-6 expression, and p300 was also identified as a USP24 substrate in our recent study^31^. Therefore, the level of p300 in M2 macrophages was assessed after USP24 knockdown (Fig. 4a). The levels of p300 and NF-κB in M2 macrophages were decreased after USP24 knockdown. Knockdown of p300 in M2 macrophages decreased NF-κB and *IL-6* mRNA levels and secreted IL-6 levels in M2 macrophages and the conditioned medium, respectively (Fig. 4b, c, j). p300 is an acetyl transferase that regulates epigenetic histones marks to regulate gene expression^32,33^. Therefore, the acetylation of histone H3 within the *NF-κB* and *IL-6* promoter regions was studied after USP24 knockdown (Fig. 4d). The silencing of USP24 in M2 macrophages decreased histone 3 acetylation at the promoter regions of *NF-κB* and *IL-6*, implying that *NF-κB* and *IL-6* transcription were inhibited after USP24 knockdown. Because our previous study confirmed that p300 is a USP24 substrate in lung cancer cells^31^, we wanted to confirm whether p300 is also directly regulated by USP24 in M2 macrophages. Indeed, we observed that USP24 interacts with p300 (Fig. 4e), and knockdown of USP24 increased p300 ubiquitination, and this effect of USP24 knockdown was abolished after treatment with the proteasome inhibitor MG132 (Fig. 4f, g). Knockdown of USP24 also decreased the protein stability of p300 in M2 macrophages and A549 cancer cells (Fig. 4h and Supplementary Fig. 3a). In addition to the epigenetics modifications placed by p300, previous studies have also indicated that several transcription factors, such as NF-κB, AP-1, Stat3, CEBP and CREB, activate the *IL-6* promoter^34,35^. Herein we used western blotting and Q-PCR to show that the NF-κB protein and mRNA levels, but not the protein levels of other factors, such as c-Jun, Stat3, CREB, and CEBP, were decreased in M2 macrophages after USP24 knockdown (Fig. 4i, j and Supplementary Fig. 3b). Knockdown of p300 decreased the protein and mRNA levels of *NF-κB* (Fig. 4k, l). Overexpression of HA-p300 abolished the effect of USP24 knockdown on *IL-6* expression (Fig. 4m). Finally, NF-κB was recruited to the promoter region of *IL-6*, and USP24 knockdown and p300 knockdown decreased the interaction between NF-κB and the *IL-6* promoter (Supplementary Fig. 3c, d). These results indicate that USP24 may upregulate *IL-6* expression through directly stabilizing p300 and indirectly induce *NF-κB* expression through p300 in M2 macrophages.

### USP24 increases *IL-6* expression by epigenetic regulation

**Fig. 5:**
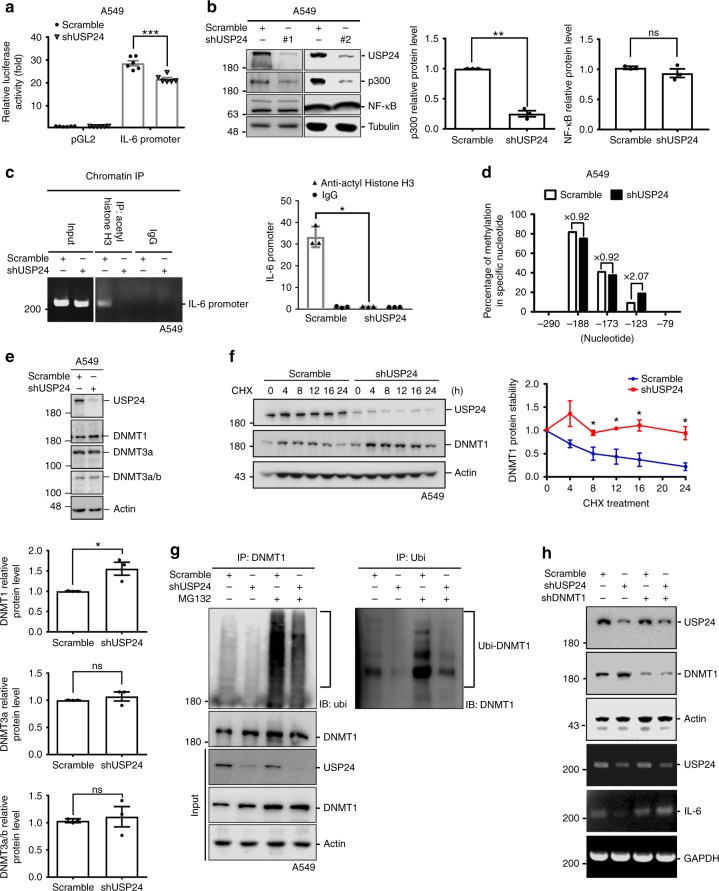
Methylation status of *IL-6* promoter and DNMT1 level in USP24-knockdown A549 cells. **a** Reporter assay was performed to analyze *IL-6* promoter activity in A549 cells after scramble knockdown or USP24 knockdown (*n* = 6). **b** Western blotting was used to analyze indicated protein level in scramble and USP24-knockdown A549 cells by using two different shRNA clones (*n* = 3). **c** Recruitment of acetyl histone H3 to the promoter of *IL-6* in scramble- and USP24-knockdown A549 cells was analyzed by chromatin immunoprecipitation assay. Anti-acetyl histone H3 antibody was used and *IL-6* promoter was analyzed by RT-PCR and Q-PCR (*n* = 3). **d** Bisulfite sequencing was used to analyze methylation sites in between −375 and −19 region. PCR products were inserted into yT&A vectors and amplified with competent cells. Sequences of the colonies derived from these competent cells were analyzed by Mission Biotech. Methylation status of each site analyzed by bisulfite sequencing in A549 cells was represented in percentage. **e** DNMT1, DNMT3a, and DNMT3a/b levels were analyzed in scramble- and USP24-knockdown A549 cells by western blotting (*n* = 3). **f** Scramble- and USP24-knockdown A549 cells were treated with CHX and collected at indicated time points. DNMT1 protein stability was analyzed by western blotting (*n* = 3). **g** USP24 was knockdown in A549 cells, and then cells were harvested for immunoprecipitation assay with anti-DNMT1 and anti-Ubi antibodies. IP samples were analyzed by western blotting with antibodies against the indicated proteins. **h** Samples collected from A549 cells with or without knockdown of USP24 and DNMT1 were used to study the mRNA levels of *USP24*, *IL-6*, and *GAPDH* by using RT-PCR. Results of western blot and Q-PCR were normalized with actin, tubulin or reads of scramble-IgG and expressed as fold of control. Data are shown as mean ± SEM, two-tailed unpaired Student’s *t*-test, ns for not significant, *P *<* 0.05, **P < 0.01, and ***P < 0.005

Because IL-6 in the tumor-associated microenvironment is not only expressed by M2 macrophages but also by cancer cells themselves^29,30^, we assessed IL-6 levels in lung cancer cells after USP24 knockdown (Fig. 5). In lung cancer cells, the *IL-6* promoter activity was decreased after USP24 knockdown (Fig. 5a). Analysis of p300 and NF-κB levels in cancer cells showed that p300 but not NF-κB was decreased after USP24 knockdown, implying that there are distinct mechanism(s) regulating *IL-6* expression in M2 macrophages and lung cancer cells (Fig. 5b). Because p300 was decreased in USP24-knockdown lung cancer cells, the histone H3 acetylation level in the *IL-6* promoter was also examined after USP24 knockdown in A549 cells (Fig. 5c). The results showed that silencing USP24 in lung cancer cells decreased histone 3 acetylation at the *IL-6* promoter region, implying that the transcriptional activity of *IL-6* is inhibited by USP24 knockdown. In addition to p300 regulation, a previous study reported that DNA promoter methylation negatively regulates *IL-6* expression^20,36^. Therefore, the methylation marks in control and USP24-knockdown M2 macrophages and A549 lung cancer cells were investigated (Fig. 5d and Supplementary Fig. 4a). After analyzing the five CpG sites upstream of the transcription start site, we found that the methylation status of a CpG site located at −123 was increased in A549 cells but not in M2 macrophages after USP24 knockdown, indicating that a decrease in *IL-6* methylation induced by USP24 in A549 cells, but not in M2 macrophages, increases *IL-6* mRNA levels. Because DNA methyltransferases (DNMTs) family members are involved in DNA methylation^37^, the DNMT1 and DNMT3a/3b levels were evaluated in USP24 knockdown A549 cells. Interestingly, the DNMT1 level was increased in A549 cells, but not in M2 macrophages, after USP24 knockdown by increased DNMT1 protein stability (Fig. 5e, f and Supplementary Fig. 4b). DNMT1 ubiquitination was also decreased in USP24-knockdown A549 cells (Fig. 5g), indicating that USP24 stabilizes the crucial modulators that negatively regulate DNMT1, resulting in the upregulation of DNMT1 after USP24 knockdown. Finally, we found that knockdown of DNMT1 in USP24-knockdown A549 cells can rescue *IL-6* level, suggesting that USP24-mediated DNMT1 degradation is really involved in *IL-6* expression (Fig. 5h).

### USP24 stabilizes β-TrCP in lung cancer cells

**Fig. 6:**
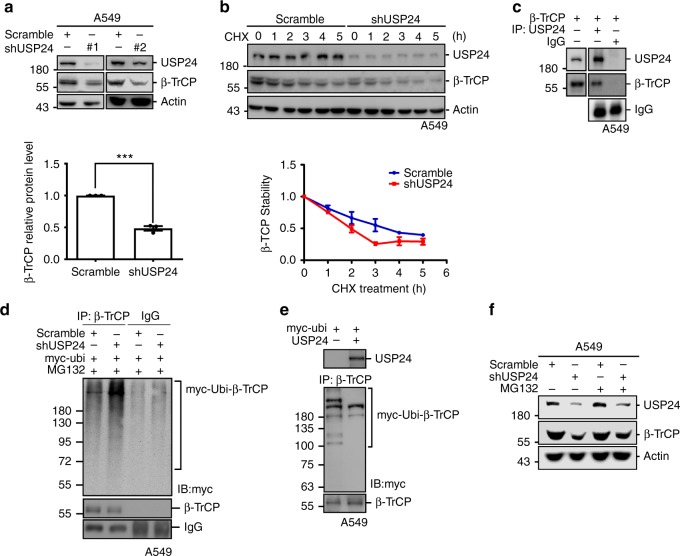
USP24 stabilizes β-TrCP in A549 cells. **a** Western blotting was used to analyze β-TrCP level in scramble knockdown and USP24 knockdown A549 cells by using two different shRNA clones (*n* = 3). **b** Scramble- and USP24-knockdown A549 cells were treated with CHX and β-TrCP protein stability was analyzed by western blotting (*n* = 3). **c** β-TrCP overexpressed A549 cells were collected for immunoprecipitation with anti-USP24 antibody and indicated proteins were analyzed by western blotting. **d** Scramble-knockdown and USP24-knockdown A549 cells were overexpressed with myc-ubi and collected for immunoprecipitation with anti-β-TrCP antibody. Indicated protein was analyzed by western blotting. **e** A549 cells were overexpressed with myc-ubi and harvested for immunoprecipitation with anti-β-TrCP antibody. Human purified USP24 protein was mixed with IP sample and analyzed by western blotting. **f** Indicated protein levels of samples from scramble or USP24 knockdown A549 cells with or without MG132 treatment were analyzed by western blotting. Results of western blot were normalized with actin and expressed as fold of control. Data are shown as mean ± SEM, two-tailed unpaired Student’s *t*-test, ***P < 0.005

To investigate how USP24 regulates DNMT1 stability, the levels of ubiquitin like with PHD and ring finger domains 1 (UHRF1) and β-TrCP, two DNMT1-targeting E3 ligases, were examined in USP24-knockdown lung cancer cells (Fig. 6a and Supplementary Fig. 5a). In addition, a previous study reported that the acetyltransferase Tip60 increases DNMT1 degradation by increasing DNMT1 acetylation and interaction with its E3 ligase^38^. Therefore, Tip60 level was also examined in USP24-knockdown A549 cells (Supplementary Fig. 5b). The results showed that UHRF1 and Tip60 levels were not affected by USP24 knockdown; however, β-TrCP was significantly decreased in USP24-knockdown A549 cells (Fig. 6a). Based on these results, we used cycloheximide treatment to examine β-TrCP protein stability and found that β-TrCP stability was decreased after USP24 knockdown (Fig. 6b). Because USP24 is a DUB, we clarified whether USP24 stabilizes β-TrCP by targeting and removing β-TrCP ubiquitin signals. Immunoprecipitation analysis confirmed the interaction between USP24 and β-TrCP (Fig. 6c), and ubiquitinated β-TrCP was significantly increased in USP24-knockdown A549 cells (Fig. 6d and Supplementary Fig. 5c). To further clarify whether USP24 directly targets β-TrCP for deubiquitination, an in vitro deubiquitination assay was performed, and purified human USP24 significantly decreased the β-TrCP ubiquitination signal (Fig. 6e). Treatment with the proteasome inhibitor MG132 could reverse the effect of USP24 knockdown in β-TrCP (Fig. 6f). Although the actual ubiquitinated lysine residues of β-TrCP were not yet identified, previous studies predicted several putative lysine residues in positions 11, 14, 55, 304, 315, and 449 of β-TrCP could be targeted for ubiquitination^39,40^. By mutating these suspected ubiquitinated lysine residues, we found that Lys 304 might be one of the ubiquitination site responsible for its protein stability, but the detail ubiquitination sites within β-TrCP still need to be addressed in the future (Supplementary Fig. 5d). These results provide evidence that USP24 directly deubiquitinates and stabilizes β-TrCP.

### USP24 decreases DNMT1 and IκB through stabilizing β-TrCP

**Fig. 7:**
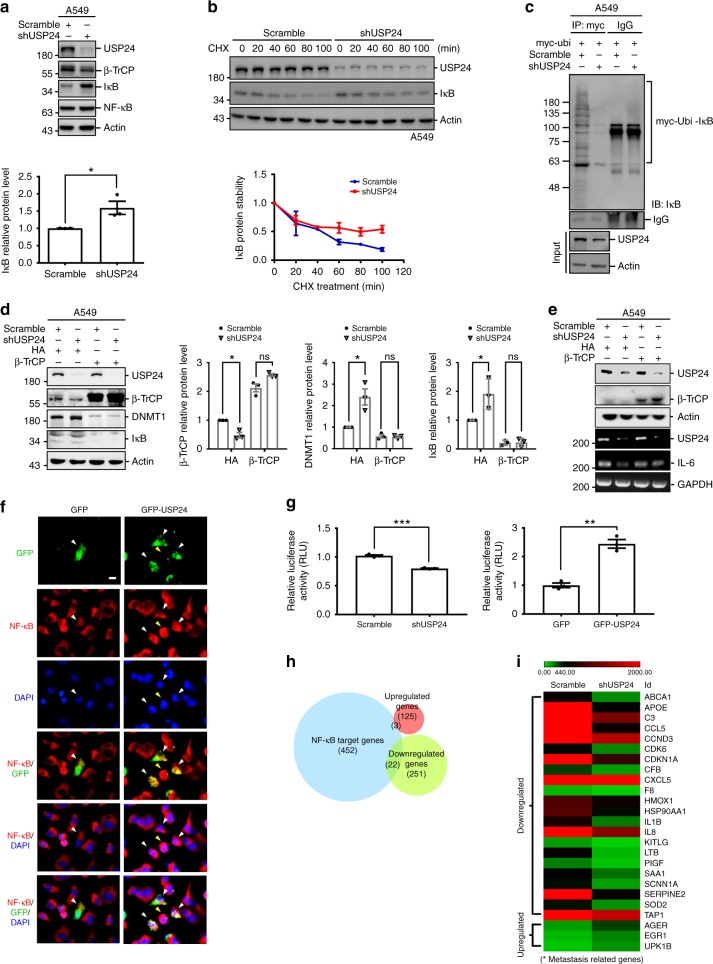
The role of β-TrCP in USP24 knockdown increased DNMT1 and IκB. **a** Indicated protein levels were examined in scramble knockdown and USP24-knockdown A549 cells by western blotting (*n* = 3). **b** Scramble- and USP24-knockdown A549 cells were treated with CHX and IκB protein stability was analyzed by western blotting (*n* = 3). **c** Scramble- and USP24-knockdown A549 cells were overexpressed with myc-ubi and cells were collected for immunoprecipitation with anti-myc antibody. IP samples were analyzed by western blotting. **d** Scramble-knockdown and USP24-knockdown A549 cells were overexpressed with β-TrCP and collected for analyzing indicated proteins by western blotting (*n* = 3). Results of western blot were normalized with actin and expressed as fold of control. **e** Indicated proteins and mRNAs levels in A549 cells with or without USP24 knockdown or β-TrCP overexpression were analyzed by western blotting and RT-PCR. **f** GFP and GFP-USP24 overexpressed A549 cells were fixed for immunofluorescence assay with indicated antibodies. GFP or GFP-USP24 overexpressed cells were indicated with white arrowheads. Yellow arrowheads indicate GFP-USP24 negatively expressed cells in GFP-USP24 overexpression group. Scale bar represents 10 μm. **g** Luciferase activity was measured by transfecting NF-κB response element (RE) containing vector into scramble knockdown, USP24 knockdown, GFP overexpressed, and GFP-USP24 overexpressed A549 cells (*n* = 3). Results were normalized with control and expressed as fold of control. **h**, **i** The intersections between 452 NF-κB target genes (blue circle), 125 USP24 upregulated genes (red circle) and 251 USP24 downregulated genes (green circle) were analyzed (**h**). Heatmaps of intersected were shown, and metastatic genes were indicated by asterisks (**i**). Data are shown as mean ± SEM, two-tailed unpaired Student’s *t*-test, ns for not significant, *P < 0.05 and ***P < 0.005

Although the NF-κB level was not affected in USP24-knockdown lung cancer cells, the protein level and stability of IκB, a well-known inhibitor of NF-κB, was significantly increased (Fig. 7a, b)^41^. Ubiquitinated IκB was also significantly decreased in USP24-knockdown A549 cells (Fig. 7c), suggesting that USP24 regulates IκB by affecting protein degradation. Nevertheless, previous studies have reported that IκB is also a substrate of β-TrCP^42,43^, suggesting that β-TrCP might be the crucial factor driving USP24 knockdown-induced IκB upregulation. β-TrCP overexpression in USP24-knockdown cells prevented the upregulation of DNMT1and IκB induced by USP24 knockdown and demonstrated that β-TrCP is critical in the USP24-induced increase of DNMT1 and IκB expression (Fig. 7d). In addition, β-TrCP overexpression can abolish the effect of USP24 knockdown in *IL-6* expression (Fig. 7e). IκB is a well-known interaction partner of NF-κB and inhibits the transcriptional activity of NF-κB by preventing its nuclear translocation^44,45^. We next clarified whether USP24 knockdown-induced IκB upregulation inhibited NF-κB nuclear translocation. Immunofluorescence analysis revealed that the nuclear translocation of NF-κB was increased in GFP-USP24-overexpressing cells (Fig. 7f). To further examine the activity of NF-κB in USP24-knockdown cells, a reporter assay was performed with a vector encoding the NF-κB response element. The promoter activity of the vector containing the NF-κB response element was downregulated in USP24-knockdown cells and upregulated in GFP-USP24-overexpressing cells (Fig. 7g). Furthermore, previous cDNA microarray data revealed that many NF-κB target genes were affected in USP24-knockdown A549 cells, and that many of these target genes were downregulated (Fig. 7h). After further analysis of the NF-κB target genes affected by USP24 knockdown, we found that several metastasis-related genes were downregulated in USP24-knockdown A549 cells^4^; these genes include *IL-1B* (Interleukin 1 beta), *SERPINE2* (Serpin family E member 2), *CCL5*, *HMOX1*(Homo sapiens heme oxygenase 1), *SAA1* (Serum amyloid A protein 1), *IL-8*, and *APOE* (Apolipoprotein E) (Fig. 7i). These findings further implied that NF-κB is indeed involved in USP24-promoted metastasis.

**Fig. 8:**
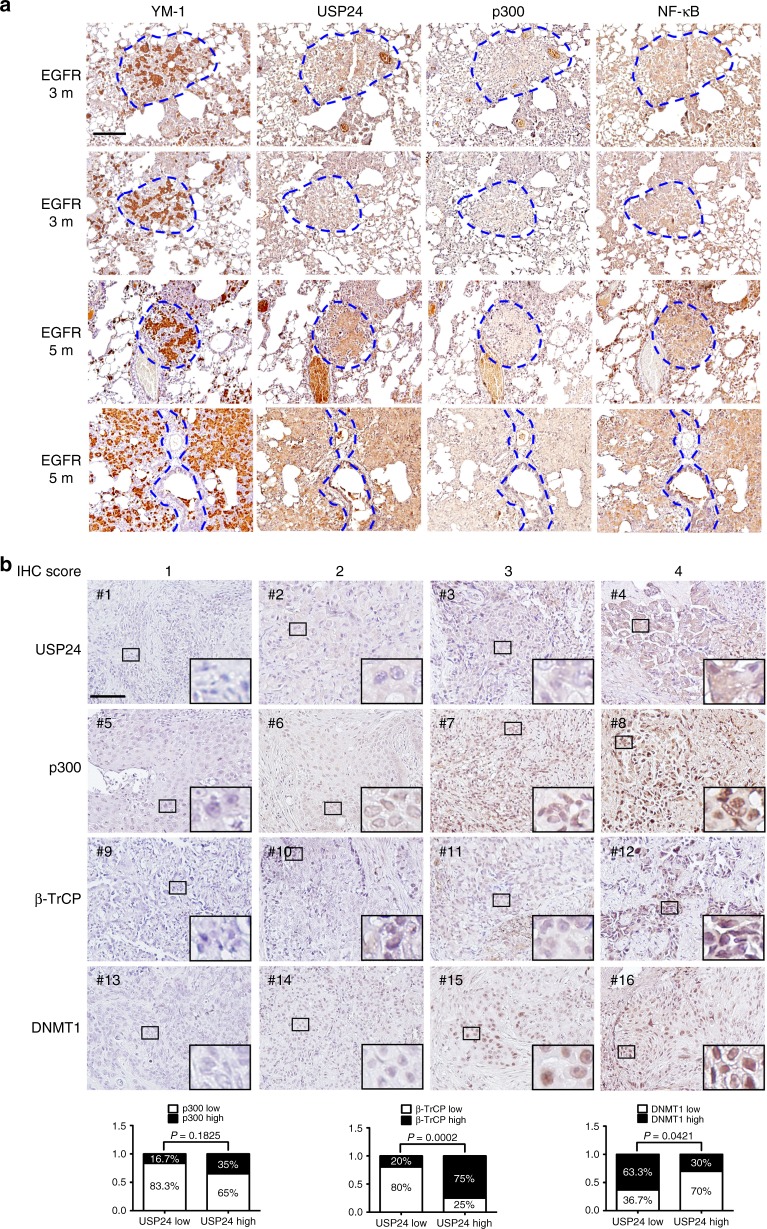
Correlation between M2 macrophage marker, USP24, p300, NF-κB, β-TrCP, and DNMT1 in lung cancer specimens. **a** Eight weeks old of EGFR^L858R^ transgenic mice were treated with doxycycline for 3 months or 5 months to induce the formation of lung cancer, and then sacrificed at indicated time points. Lungs were harvested and immunohistochemistry was performed to analyze the expression of indicated proteins. YM-1 positive area was marked with blue dashed lines. Scale bar represents 50 μm. **b** Immunohistochemistry staining was used to analyze indicated protein in 50 human lung cancer specimens. Each slide was given with different scores to indicate the intensity of protein signals. Samples with score 1 and 2 were considered as low protein expression, and score 3 and 4 were considered as high protein expression. Scale bar represents 50 μm. In slides stained with anti-USP24 antibody, 30 samples were identified as low expression, and 20 samples were identified as high expression. Expression level of indicated proteins in low USP24 expression groups or high USP24 expression groups were shown in percentage. Correlation between USP24 expression and p300, β-TrCP, and DNMT1 were examined by Fisher’s exact test. *P* value of the correlation of USP24 to p300, USP24 to β-TrCP, and USP24 to DNMT1 were 0.1825, 0.0002, and 0.0421, respectively

There are different regulatory mechanisms controlling USP24-induced *IL-6* expression in M2 macrophages and lung cancer cells. We collected lung cancer samples from a doxycycline-induced lung cancer mouse model to study the correlation among USP24, p300 and NF-κB in M2 macrophages in vivo. EGFR^L858R^ mice were fed doxycycline for 3 or 5 months to induce adenocarcinoma formation, and lung cancer tissues were analyzed using IHC staining. All the antibodies used were proven to have high specificities for the indicated proteins in the samples from mice and human cohorts (Supplementary Fig. 6a, b). USP24, p300, and NF-ĸB were highly expressed in cells positive for YM-1 expression, which is an M2 macrophage marker^46^, providing evidence that the USP24-p300-NF-ĸB signaling pathway was expressed in this in vivo model (Fig. 8a). Next, we collected 50 human lung cancer specimens to study the correlation among USP24, p300, β-TrCP and DNMT1 in human lung cancer (Fig. 8b). The signals from each protein were evaluated and assigned different scores to indicate the intensity of protein expression observed on the IHC slides after the validating the specificity of the indicated antibodies (Supplementary Fig. 6b). Samples with scores of 1 and 2 were considered low expression, whereas scores of 3 and 4 were considered high expression (Fig. 8b). The results showed a positive correlation between USP24 and p300 and between USP24 and β-TrCP. A negative correlation between USP24 and DNMT1 was observed in human lung cancer specimens. These findings further support the regulatory role of USP24 and the detailed mechanism reported in this study.

## Discussion

**Fig. 9:**
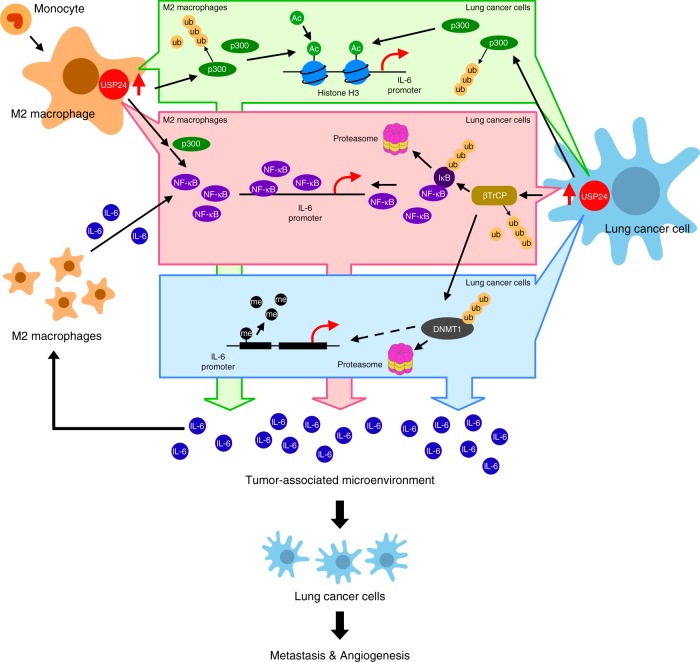
Schematic summary of the role of USP24 in IL-6 mediated metastasis and angiogenesis. USP24 promotes *IL-6* expression by increasing the levels of NF-κB and histone H3 acetylation through stabilizes p300 in the M2 macrophages. In lung cancer cells, USP24 increases p300 and β-TrCP levels which promotes histone H3 acetylation, NF-κB nuclear translocation and decreases *IL-6* promoter methylation, thus resulting in the upregulation of IL-6 level in tumor-associated microenvironment and lung cancer malignancy

In this study, different mechanistic details regarding USP24-mediated *IL-6* expression were observed in M2 macrophages and cancer cells. In M2 macrophages, USP24 increased p300 levels, subsequently enhancing the levels of NF-κB and IL-6. In cancer cells, USP24 increased p300 and β-TrCP, thus increasing the acetylation of histone H3 and the degradation of DNMT1 and IκB, resulting in the recruitment of histone H3 acetylation to the promoter region of IL-6, the reduction of DNA methylation, and the promotion of NF-κB nuclear translocation, thereby facilitating *IL-6* expression (Fig. 9).

The tumor-associated microenvironment is critical for cancer progression, including cancer formation and malignancy^6,47,48^. Various cell types, such as immune cells, fibroblasts, bone marrow-derived inflammatory cells, lymphocytes, and adipocytes, as well as the extracellular matrix, establish the tumor-associated microenvironment and can be affected by various factors released by tumor cells^49,50^. In this study, USP24 expression was increased not only in lung cancer cells but also in M2 macrophages. While studying the role of USP24 upregulation in M2 macrophages, we found that USP24 expression in M2 macrophages enhanced cancer metastasis by inducing *IL-6* expression. Our previous study also showed that USP24 was increased in lung cancer cell lines with higher metastasis activity^4^. Here, we also observed that USP24 in lung cancer cells increased *IL-6* expression. Therefore, IL-6 in the tumor-associated microenvironment might be derived from both M2 macrophages and cancer cells based on this study. However, whether USP24 levels are also affected in other cell types such as fibroblasts and adipocytes within the tumor-associated microenvironment remains unknown and requires future clarification. In addition, IL-6 is not the only factor regulated by USP24 in M2 macrophages; the data in the Supplementary Fig. 2b, c, and previous studies indicated that IL-8 is also involved in cancer progression^51,52^, indicating that other factors regulated by USP24 might affect cancer progression. Until recently, few studies have examined the role(s) of USPs in the tumor-associated microenvironment. However, several studies have reported a relationship between DUBs and inflammation. For example, USP25 negatively regulates IL-17-mediated signaling and inflammation^53^. In addition, silencing USP22 suppresses high glucose-induced apoptosis, ROS production, and inflammation in podocytes^54^. This study is the first to address the role of DUBs in tumor-associated microenvironment and its effect on cancer progression.

In this study, another interesting point is the discovery of distinct molecular mechanisms regulating *IL-6* expression in M2 macrophages and lung cancer cells in response to USP24. Previous studies examining IL-6 regulation indicated that the level of DNA methylation in the *IL-6* promoter regulates transcriptional activity in cancer cells^20,21,36^. In addition, NF-κB is recruited to the *IL-6* promoter to increase *IL-6* transcriptional activity in cancer cells^12^. Interestingly, we found that USP24 stabilizes p300 to manipulate the acetylation level of histone 3 in the *IL-6* promoter region in M2 macrophages and lung cancer cells. However, an increase in p300 by USP24 enhances the NF-κB level through an increased recruitment of acetyl histone H3 to the promoter of *NF-κB* to regulate *IL-6* expression in M2 macrophages but not in lung cancer cells. Nevertheless, the decreased IκB and *IL6* promoter methylation levels to regulate IL6 expression were observed only in lung cancer cells, implying different regulatory mechanism(s) controlling IL6 expression in M2 macrophages and lung cancer cells. Because NF-κB is important for inflammation, USP24-induced *NF-κB* expression might regulate not only *IL-6* but also the expression of many inflammation-related genes^55,56^. The cDNA microarray data revealed that many NF-κB target genes were also downregulated in USP24-knockdown lung cancer cells, and several of the genes were also found to be related to metastasis. However, many NF-κB target genes remained unaffected after USP24 knockdown, likely because other transcriptional factors involved in the transcription of these genes are also regulated by USP24. For example, a previous study indicated that NF-κB activation increased the expression of CCL2, which is involved in immune surveillance^57^. However, in this study, USP24-activated NF-κB did not increase CCL2, implying that other factors regulated by USP24 might negatively regulate CCL2 expression. In addition, we found that USP24 stabilized p300 in M2 macrophages and lung cancer cells to regulate cancer progression. p300 as an acetyl transferase regulates the expression of many genes, which might broadly affect the cancer progression. Previous studies have revealed that p300, as a tumor suppressor, inhibits cancer tumorigenesis^58^. However, other studies have also shown that p300 overexpression induces cancer malignancy^59^. In this study, we studied the effect of USP24 in lung cancer malignancy through the USP24/p300/ NF-κB/IL-6 regulatory axis. Adding the physiological dose of IL-6 to the media of USP24-knockdown cells can rescue the effect of USP24 knockdown, suggesting that the USP24-mediated IL-6 in M2 macrophages and in lung cancer is an important factor that affects USP24-induced lung cancer malignancy. In Supplementary Fig. 1e, increased tumor volume was found when CL1–5 cells mixed with M2 macrophage. Previous studies reported that NF-κB activation in macrophages increase the expression of cytokines such as TNF-α and IL-6, in turn promoting the proliferation of gastric cancer cells^60,61^. Although the increased tumor volume could cause increased metastatic burden of intravasation. To further confirm the regulatory role of USP24 in IL-6 mediated metastasis in vivo, CL1–5 cells pretreated with USP24-knockdown conditioned medium were injected into SCID mice through tail vein to clarify the ability of extravasation in these pretreated circulating tumor cells. The result shown in Fig. 3s indicated that reduced lung nodule number was found in USP24-knockdown cancer cells. Therefore, our in vivo data suggested that USP24 regulated *IL-6* expression not only affect the intravasation but also the extravasation of cancer cells.

In addition to the p300 stabilization induced by USP24 in cancer cells to regulate *IL-6* expression through increased histone H3 acetylation, USP24 also reduces DNA methylation in the promoter region of *IL-6* in lung cancer cells, but not in M2 macrophages, to increase *IL-6* transcription by decreasing DNMT1 protein stability. According to previous studies, DNMT1 is important for regulating gene expression through DNA methylation, and many genes related to the various diseases, such as cancer, have been reported to be regulated by DNMT1. Therefore, an understanding of the molecular mechanism(s) involved in the regulation of DNMT1 is critical for disease prevention^62–65^. Several studies have reported how DNMT1 protein stability is regulated by the E3 ligases, UHRF1, and β-TrCP, and the DUBs USP7^66,67^. In addition, a previous study indicated that Tip60, an acetyltransferase, increases DNMT1 degradation by increasing DNMT1 acetylation and its interaction with its E3 ligase^38^. In this study, we observed that USP24 increased DNMT1 degradation in cancer cells, but not in M2 macrophages, leading to decreased DNA methylation. Because USP24 is a DUB, we expect that DNMT1 is not a substrate of USP24. We propose that the E3 ligase(s) for DNMT1 or a protein that can increase DNMT1 degradation, such as Tip60, might be the USP24 substrate. Based on these results, β-TrCP, but not UHRF1 or Tip60, is stabilized by USP24 and regulates *IL-6*. Notably, a previous study reported that IL-6 accumulation was observed in wild-type transgenic mouse epidermis after UVB irradiation but was reduced in mice expressing a dominant-negative β-TrCP mutant^68^. In this study, we further elucidated how β-TrCP regulates *IL-6* expression and characterized a detailed regulatory pathway for USP24/β-TrCP/DNMT1/IL-6.

Immunotherapy is one of the best strategies for cancer treatment, and interactions between tumor-associated microenvironment and tumors are crucial for cancer development. Many factors in the tumor-associated microenvironment and cancer are regulated during cancer progression through different pathways, such as transcriptional regulation and protein degradation. Most previous studies have focused on transcriptional activity, and few studies have addressed the protein degradation. This study shows that USP24 mediates p300, NF-κB, DNMT1, and β-TrCP to regulate *IL-6* expression, thus affecting cancer metastasis. We believe that future studies will identify that not only transcription but also post-translational modifications are involved in the interactions between the tumor-associated microenvironment and tumor cells that regulate cancer progression.

## Methods

### Cell culture and treatment

Human lung adenocarcinoma epithelial cell line A549 and human monocyte THP-1 cell line were culture with RPMI 1640 medium (Invitrogen) containing 10% fetal bovine serum (FBS, Thermal Fisher), 100 μg per ml streptomycin and 100 U per ml penicillin G sodium (Thermal Fisher). Human microvascular endothelial cells (HMEC-1) and human umbilical vein endothelial cells (HUVECS) were culture with HiEndoXL Endothelial Cell Expansion Medium, Reduced Serum (HiMedia). All cells were incubated at 37 °C with 5% CO_2_. For the differentiation of M2 macrophages, THP-1 cells were treated with 100 μM phorbol 12-myristate 13-acetate (PMA; EMD Millipore) for 24 h, and then treated with 20 μg per ml IL-4 (Peprotech) and 20 μg per ml IL-13 (Peprotech) for another 24 h. For transfecting plasmid, Polyjet (SignaGen) was used according to manufacturer’s instructions. All cell lines were obtained from American Type Culture Collection (ATCC) and were tested for mycoplasma contamination and results were negative.

### Western blotting

Cells were collected by sample buffer and analyzed by electrophoresis. Proteins were transferred to polyvinylidene difluoride (PVDF, Millipore) membrane and TBST buffer (10 mM Tris-HCl, pH 8.0, 150 mM NaCl and 0.05% Tween 20) containing 5% nonfat milk was used for blocking. Anti-USP24 (Cat# 13126-1-AP, Proteintech, 1:3000), anti-p300 (Cat# 554215, BD, 1:2000), anti-phospho-Stat3 (Cat# 9145, Cell Signaling, 1:1000), anti-tubulin (Cat# 109832, Genetex, 1:10000), anti-NF-κB (Cat# 372, Santa Cruz, 1:200), anti-IκB (Cat#371, Santa Cruz, 1:200)anti-actin (Cat# 110564, Genetex, 1:20000), anti-ubiquitin (Cat# 9133, Santa Cruz, 1:200), anti-DNMT1 (Cat# 10222, Santa Cruz, 1:200), anti-DNMT3a (Cat# 365769, Santa Cruz, 1:200), anti-DNMT3a/b (Cat# 10234, Santa Cruz, 1:200), anti-UHRF1 (Cat# 373750, Santa Cruz, 1:200), anti-β-TrCP (Cat# 4394, Cell Signaling, 1:1000), anti-Tip60 (Cat# 166323, Santa Cruz, 1:200), anti-myc-tag (Cat# 2272, Cell Signaling, 1:2000), anti-STAT3 (Cat# 482, Santa Cruz, 1:200), anti-c-Jun (Cat# 610327, BD, 1:1000), anti-C/EBPδ (Cat# 115047, Genetex, 1:1000), anti-CREB1 (Cat# A1189, Abclonal, 1:1000), were used for probing interested proteins. After incubated with primary antibodies, PVDF membranes were then incubated with secondary immunoglobulin antibodies linked with horse radish peroxidase (Millipore, 1:10,000). For detecting immunoprecipitated samples, light chain specific secondary antibodies were used (Jackson ImmunoResearch, 1:10000). ECL Western blotting detection system (Millipore) and ChemiDoc-it imager (UVP) were used for detecting signals.

### Preparation of conditioned medium

THP-1 cells and M2 macrophages were incubated at 37 °C for 2 days and medium was collected and centrifuged at 800 rpm for 5 min. Supernatant was mixed with freshly prepared medium contained 10% FBS at 1–2 ratio for preparing conditioned medium. Scramble knockdown or USP24-knockdown M2 macrophages were washed with PBS after 24 h of lentivirus infection, and cells were incubated in fresh medium. After 72 h of incubation, medium collected from scramble knockdown or USP24-knockdown M2 macrophages were centrifuged at 800 rpm for 5 min, and supernatant was mixed with freshly prepared medium contained 10% FBS at 1 to 2 ratio for preparing conditioned medium.

### In vitro deubiquitination assay

A549 cells were overexpressed with myc-ubi and treated MG132 (Sigma-Aldrich) for 12 h. β-TrCP protein in A549 cell lysates were immunoprecipitated by incubating with anti-β-TrCP antibody for 4 h, and then incubated with protein A agarose (Millipore) for 1 h. After washing, substrate was mixed with human recombinant USP24 protein (50 μg per ml) (Origene) in deubiquitination buffer (50 mM Tris PH 8.0, 10 mM DTT and 5 μM MG132) for 2 h in 37 °C. Reaction was stopped by adding sample buffer and signal of ubiquitinated proteins were analyzed by western blotting.

### Lentivirus knockdown system

Scramble knockdown, USP24 knockdown and p300 knockdown lentivirus were generated from RNAi core facility of Academia Sinica (Taiwan). Cells were seeded in 6-well plates and incubated for 16 h, and then treated with 1 ml RPMI medium containing 10 μg Polybrene (Millipore) and lentivirus with 5 Multiplicity of infection (MOI). After 24 h of infection, medium containing lentivirus was replaced with fresh medium and maintained for another 72 h.

### Luciferase reporter assay

8 × 10^4^ A549 cells were seeded in each well of 6-well plates for 16 h, and lentivirus were added as described. Three days after lentivirus infection, reporter plasmids containing *IL-6* promoter region or NF-κB response element (Promega) were transfected into A549 cells. Reporter assays were performed by using Dual-luciferase reporter assay system (Promega) following manufacturer’s instruction.

### Chromatin immunoprecipitation

Cells were infected with scramble, shUSP24, or shp300 shRNA expressing lentivirus for four days, and then cells were incubated with medium containing 1% formaldehyde (Sigma-Aldrich) for 10 min at room temperature for cross-linking. Cells were washed with PBS and lysed with lysis buffer (25 nM, pH 7.5 Tris-HCl, 150 mM NaCl, 5 mM EDTA, 1% Triton X-100, 1% SDS). Samples were sheared on ice by sonication (output level of 4, 15 s on, and 45 s off, total 3 min). Fifty microliter of supernatant then collected and diluted with 450 μl of dilution buffer (50 mM, pH 8.0 Tris-HCl, 0.5% NP-40, 0.2 M NaCl, 0.5 mM EDTA). Samples were incubated with 20 μg of sonicated salmon sperm DNA (Invitrogen) for 2 h at 4 °C on a rotating device, then indicated antibodies (1:200) were added and incubated for another 16 h on a rotating device. Protein A or protein G agarose beads were added and incubated for 1 h at 4 °C, and collected by centrifugation at 4000 rpm at 4 °C for 1 min and washed three times with buffer (20 mM pH 8.0 Tris-HCl, 0.5% NP-40, 0.5 M NaCl, 2 mM EDTA) and three times with buffer (10 mM pH 8.0 Tris-HCl, 0.5% NP-40, 0.1 M NaCl, 1 mM EDTA, 0.01% SDS). Beads were suspended with 500 μl of TE buffer containing 1% SDS, and boiled at 65 °C for 2 h. Supernatant was collected and heated with 65 °C for another 16 h. DNA was precipitated and washed with 70% alcohol. Indicated genes were detected by PCR. Primer sequences are listed in Supplementary Table 1.

### Chromatin fractionation

3 × 10^6^ cells were washed with cold PBS and suspended with cold buffer A (10 mM HEPES pH 7.9, 10 mM KCl, 1.5 mM MgCl_2_, 0.34 M sucrose, 10% glycerol, 1 mM dithiothreitol) containing protease inhibitor. Suspended cells then treated with 0.1% Triton X-100 at 4 °C for 5 min. Cytoplasmic proteins were separated from nuclei by centrifugation at 1300×*g* for 4 min at 4 °C. Nuclei were then washed with buffer A and treated with buffer B (3 mM EDTA, 0.2 mM EGTA, 1 mM dithiothreitol) containing protease inhibitor on 4 °C for 30 min. Soluble nuclear proteins were separated from insoluble chromatin by centrifugation at 1700×*g* for 4 min. Insoluble chromatin was washed buffer B and then suspended with buffer A. Total cell lysate, cytoplasmic proteins, soluble nuclear proteins, and insoluble chromatin were mixed with sample buffer and analyzed by western blotting.

### Immunohistochemistry

Human and mouse specimens were incubated in 10% formaldehyde for 72 h for fixation, dehydration, and embedded in paraffin. For immunohistochemistry, xylene (Sigma-Aldrich) was used for dewaxing paraffin-embedded sections and serial diluted ethanol was also used for dehydration. Endogenous peroxidases were blocked by incubating in PBS containing 0.3% hydrogen peroxide (Sigma-Aldrich) for 30 min, and then samples were blocked with 1% bovine serum albumin. Proteins of interest were recognized by incubated with anti-USP24 (Cat#13126-1,-AP, Proteintech, 1:200), anti-p300 (Cat#585, Santa Cruz, 1:100), anti-DNMT1 (Cat#271729, Santa Cruz, 1:100), anti-β-TrCP (Cat#102667, Genetex, 1:100), and anti-NF-ĸB (Cat#8414, Santa Cruz, 1:100) at room temperature for 3 h, and immunoreactivity was visualized by using Vectastain ABC kit (Vector). Sections were photographed by Olympus BX-51 microscope. Specificities of the antibodies for indicated proteins were validated by using the samples from mice and human cohorts and uncropped scans of the blots were shown in Supplementary Fig. 5.

### Immunofluorescence

Cells were infected with scramble or shUSP24-knockdown lentivirus, and seeded in 6-well plates with cover slip inside for 24 h. Cells on cover slips were fixed with PBS containing 4% paraformaldehyde at 4 °C for 15 min. After washing with PBS, cover slips were incubated with PBS containing 0.2% Triton X-100 for 5 min at room temperature. PBS containing 1% bovine serum albumin was used for blocking cover slips for 1 h, and indicated antibodies were used for staining for 16 h at 4 °C. After washing with PBS, cells were stained with Alexa Fluor® 488 or 568 (Invitrogen) for 1 h at room temperature. Cells were then stained with DAPI (Invitrogen) and cover slips were fixed on glass slides. Signals of indicated proteins or DAPI were detected by fluorescence Olympus BX-51 microscopy.

### Transwell migration assay

A549 or CL1–5 cells were treated with indicated conditioned medium with or without IL-6 for 24 h. 2 × 10^4^ cells were suspended with serum-free medium and seeded into insert of transwell chamber (Costar). Lower chambers were filled with medium containing 10% FBS, and cells were incubated at 37 °C. After 16 h of incubation, migrated cells were fixed with methanol and stained with DAPI. Image of migrated cells were taken by fluorescence Olympus BX-51 microscopy and migrated cells number were analyzed by ImageJ.

### Chemotaxis assay

A549 or CL1–5 cells were suspended with serum free medium and seeded into insert of transwell chamber, and lower chambers were filled with conditioned medium with or without IL-6. After 16 h of incubation, migrated cells were fixed with methanol and stained with DAPI. Image of migrated cells were taken by fluorescence Olympus BX-51 microscopy and migrated cells number were analyzed by ImageJ.

### Wound-healing migration assay

A549 cells were maintained in 6 cm dishes till reaching 80% density, and scratched with 200 μl tips. Cells were washed with PBS and photographed under microscopy observation. Cells then treated with indicated conditioned medium and photographed again after 24 h of treatment. Migrated distance then was measures and relative migrated distance was analyzed.

### In vitro angiogenesis assay

ECMatrix (Millipore) was mixed with dilution buffer and added into 96-well plates. Plates containing ECMatrix mixture were incubated at 37 °C for 1 h. 2 × 10^4^ of human microvascular endothelial cells (HMEC-1) and human umbilical vein endothelial cells (HUVEC) were harvested and mixed with 150 μl of conditioned medium and then seeded into each well of 96-well plates. Cells were incubated for 12 h at 37 °C. Tubule formation was observed under microscopy.

### Cytokine array

Conditioned medium was collected from scramble knockdown or USP24 knockdown M2 macrophages and analyzed by Proteome Profiler Human XL Cytokine Array Kit (R&D Systems). Experiments were performed according to the manufacturer’s protocols. Quantitative analysis of blotting spot was performed by using Multi Gauge software (FUJIFILM).

### Xenograft animal model

CL1–5 cells, and scramble, or shUSP24 knockdown lentivirus infected M2 macrophages were harvested. 2 × 10^6^ CL1–5 cells, 1 × 10^6^ CL1–5 cells mixed with 1 × 10^6^ scramble knockdown M2 macrophages, and 1 × 10^6^ CL1–5 cells mixed with 1 × 10^6^ USP24-knockdown M2 macrophages were prepared and suspended in PBS. Cells were seeded into the back of 8 week-old female SCID mice once a week (scramble group, *n* = 1; shUSP24 group, *n* = 1). After three times of injection and two months of incubation, mice were sacrificed. Tumor size and weight were measure, and hepatic angiogenesis was observed. For in vivo metastasis assay, CL1–5 cells were treated with conditioned medium derived from scramble, or USP24 knockdown lentivirus infected M2 macrophages with or without IL-6 for 24 h. Treated cells were suspended in PBS and injected into 8-week-old female SCID mice through tail vein (DMEM group, *n* = 5; scramble group, *n* = 4; shUSP24 group, *n* = 4; shUSP24 + IL-6 group, *n* = 4). Two months after injection, mice were sacrificed, and metastatic lung nodules were counted. The experimental animals were approved by the Institutional Animal Care and Use Committee at National Cheng Kung University and all relevant ethical regulations were complied.

### RT-PCR and Q-PCR

RNA from indicated cells was extracted by using TRIsure RNA extraction kit (Bioline), and 3 μg of purified RNA was converted into cDNA by reverse transcription with SuperScript III reverse transcriptase (Invitrogene). PCR was performed by using SuperTherm Taq DNA polymerase (GeneCraft) according to manufacturer’s instructions. For Q-PCR analysis, SYBR Green PCR Master Mix (Thermal Fisher) was used according to manufacturer’s instructions. All values were normalized with internal control, *GAPDH*, and relative gene expression levels were then calculated. Primer sequences are listed in Supplementary Table 1.

### Bisulfite sequencing assay

Genomic DNA from scramble or USP24 knockdown A549 cells was purified by using QIAamp DNA mini kit (QIAGEN). Methylation status of purified DNA was analyzed by EZ DNA methylation kit (Zymo research) and bisulfite conversion was performed according to manufacturer’s instruction. 240 to 82 nucleotide upstream transcription start site was reported the core region of *IL-6* promoter^21^. Sequences containing these interested CpG sites were amplified by PCR, and primers were choosing by MethPrimer. Forward primer sequences are listed in Supplementary Table 1. After amplification, PCR product was extracted by using Gel extraction minipre system (Viogene), ligated into yT&A cloning vector (Yeastern biotech), and amplified by competent cells. 22 clones containing sequences generated from scramble knockdown A549 cells and 16 clones containing sequences generated from USP24 knockdown A549 cells were picked for sequencing. 8 clones containing sequences generated from scramble knockdown M2 macrophages and 9 clones containing sequences generated from USP24 knockdown M2 macrophages were picked for sequencing. Sequences of each clone then analyzed by Mission Biotech.

### Single mutation

The Lys residues of β-TrCP were mutated to arginine by using a polymerase chain reaction (PCR) mutagenesis method by following manufacturer’s instructions (Stratagene). Primer sequences for single mutation are listed in Supplementary Table 1.

### ELISA

Hunab EKISA Ready-SET-GO kit (eBioscience) was used by following manufacturer’s instructions for measuring secreted IL-6 level.

### Intersection of NF-κB target genes and USP24 regulated genes

452 NF-κB target genes^69,70^, 125 USP24 upregulated genes and 251 USP24 downregulated genes were analyzed. Heatmaps of 25 intersected genes between NF-κB target genes and USP24 regulated genes were shown, and metastatic genes were indicated by asterisk.

### Transgenic mice

Transgenic mice were acquired from Jackson Lab (Bar Harbor, MA, USA) and maintained at the National Laboratory Animal Center (NLAC), Tainan, Taiwan. The TetO-EGFR^L858R^ transgenic mice expressed EGFR^L858R^ was under the regulation of a tetracycline-responsive promoter element (TRE; tetO). EGFR^L858R^ were crossed with Scgb1a1-rtTA transgenic mice to generate bitransgenic mice. In order to induce the generation of EGFR^L858R^ in bitransgenic mice, doxycycline (0.5 g per l) was added to the drinking water, starting at the age of 8 weeks. The experimental animals were approved by the Institutional Animal Care and Use Committee at National Cheng Kung University and all relevant ethical regulations were complied.

### Specimens of human lung cancer patients

Human tissue specimens were supplied from Human Biobank, Research Center of Clinical Medicine, National Cheng Kung University Hospital, and approved by the Clinical Research Ethics Committee at National Cheng Kung University Medical Center (IRB# B-ER-104–376). Informed consent was obtained from all subjects.

### Statistics

All samples or animals (not randomized) were used for statistical analysis. The investigator was aware of the sample allocation during the experiment and when assessing its outcome for all animal experiments. For all experiments, at least three independent biological replicates of each conditions were analyzed. Estimated variation within each experiment group is similar. The difference between two groups was analyzed by two-tailed unpaired Student’s *t*-test. Correlation between USP24 expression and p300, β-TrCP, and DNMT1 in IHC data were examined by Fisher’s exact test. The *P*-value, which is < 0.05, was considered as statistically significant. Center value is defined as mean value, and s.e.m. is used to calculate and plot error bars from raw data.

## Electronic supplementary material

Supplementary Information

## Data Availability

The data that support the findings of those study are available from the corresponding author upon reasonable request.
