# Supplementary material for: USP24 induces IL-6 in tumor-associated microenvironment by stabilizing p300 and β-TrCP and promotes cancer malignancy
Source: Nat Commun. 2018 Sep 28;9:3996. doi: 10.1038/s41467-018-06178-1 (PMC6162259; doi:10.1038/s41467-018-06178-1)
Supplement: Supplementary file 1 — Supplementary Information [file 41467_2018_6178_MOESM1_ESM.pdf]

Supplementary Information

USP24 induces IL-6 in tumor-associated microenvironment by stabilizing p300 and β-TrCP and promotes cancer malignancy

Wang et al.

Supplementary Figure 1

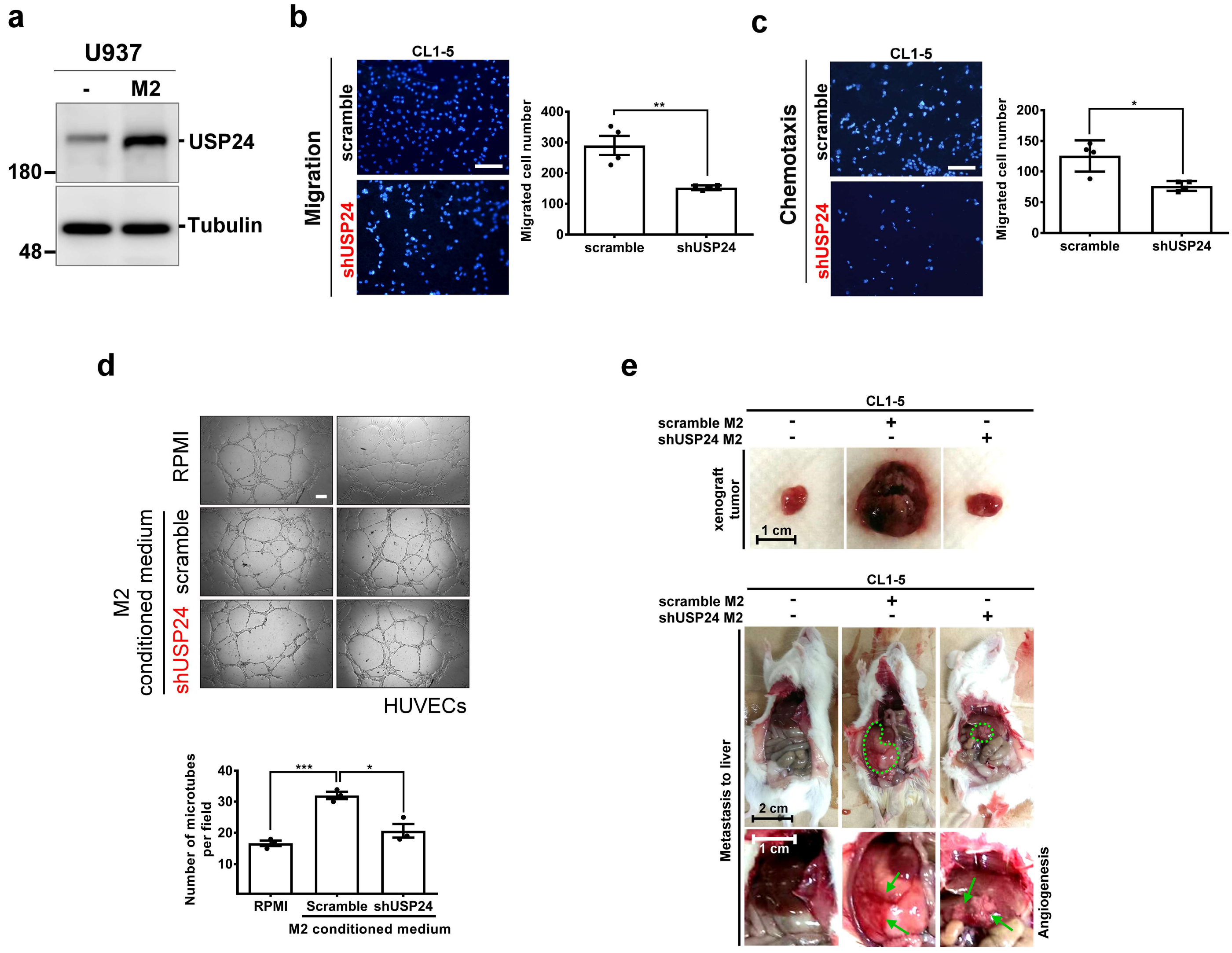

Supplementary Figure 1 – USP24 level and its pro-metastatic role in M2 macrophages. a Samples were collected from U987 monocytes and U987 derived M2 macrophages for studying the USP24 level by Western blotting. **b** CL1-5 cells were treated with conditioned medium derived from scramble knockdown or USP24 knockdown M2 macrophages for 24 hours, and transwell migration assay was performed to analyze the migratory ability of lung cancer cells (n = 4) (DAPI staining, scale bar: 100 μm). **c** Conditioned medium derived from scramble knockdown or USP24 knockdown M2 macrophages was placed in the lower chamber of transwell assay. CL1-5 cells were seeded into upper chamber and incubated for 16 hours for chemotactic assay (n = 4) (DAPI staining, scale bar: 100 μm). **d** Conditioned medium collected from M2 macrophages with or without USP24 knockdown was used to treat HUVECs cells to study the tube formation (n = 3). Scale bar represents 200 μm. **e** CL1-5 cells were mixed with scramble- or USP24-knockdown M2 macrophages and seeded on the back of SCID mice. Two months after injection mice were sacrificed and tumor volume (upper panel, scale bar: 1 cm), metastasis nodules, and angiogenesis were analyzed (middle panel, scar bar: 2 cm; lower panel, scale bar: 1cm). Hepatic metastasis and angiogenesis are indicated with green dashed lines and green arrowheads respectively. Data are shown as mean ± SEM, and two-tailed unpaired Student's t-test, ns for not significant, *P < 0.05, and ***P < 0.005.

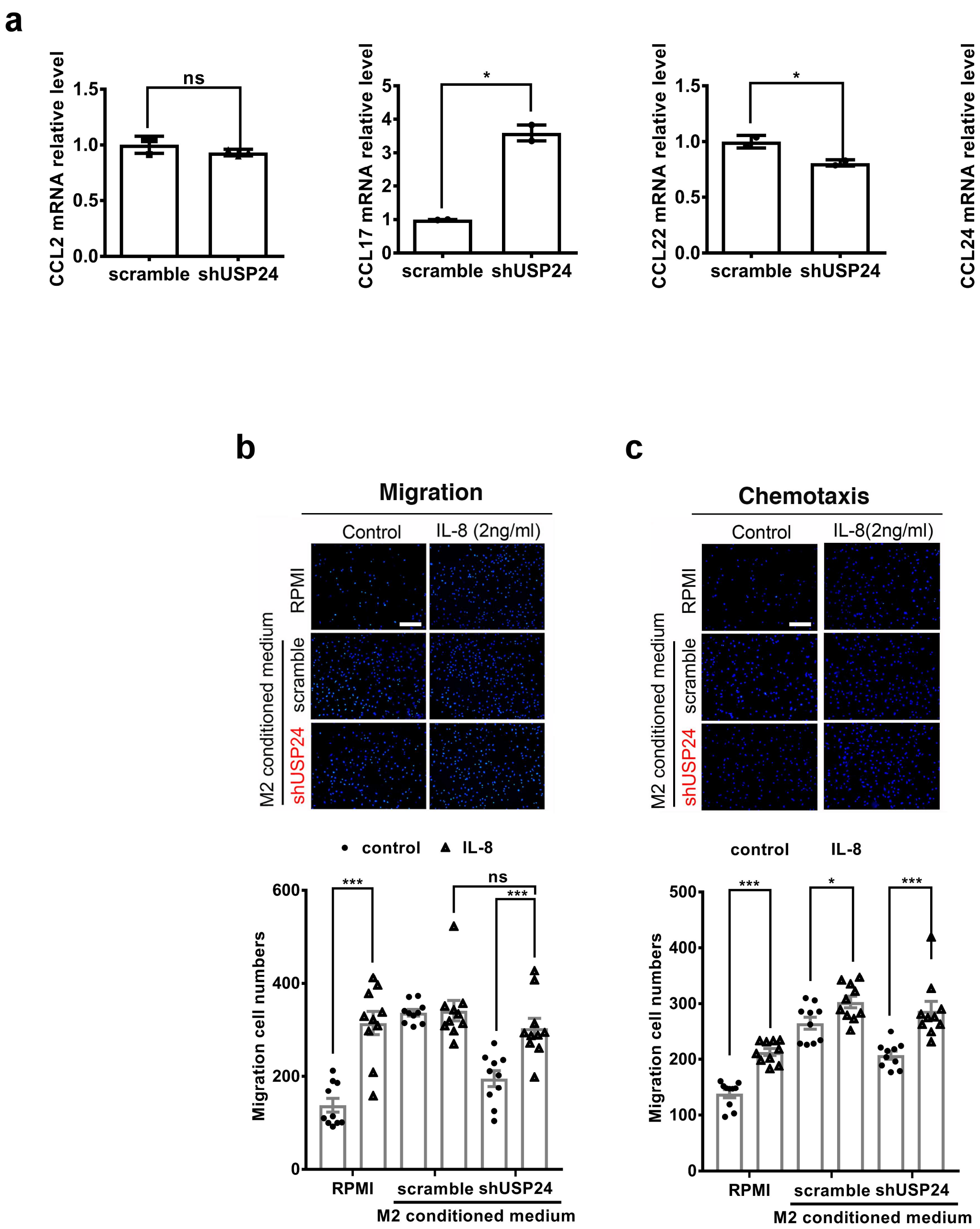

Supplementary Figure 2 – USP24 affects cytokines in M2 macrophages. a The mRNA levels of indicated cytokines from M2 macrophages with or without USP24 knockdown were studied by Q-PCR (n = 3). **b, c** A549 cells treated with RPMI, conditioned medium from scramble- or USP24-knockdown M2 macrophages with or without IL-8. Migratory ability was studied by using transwell migration assay (**b**) and chemotactic assay (**c**) (n = 10, DAPI staining, scale bar: 100 μm). Data are shown as mean ± SEM, two-tailed unpaired Student's t-test, ns for not significant, **P* < 0.05, and ****P* < 0.005.

Supplementary Figure 3

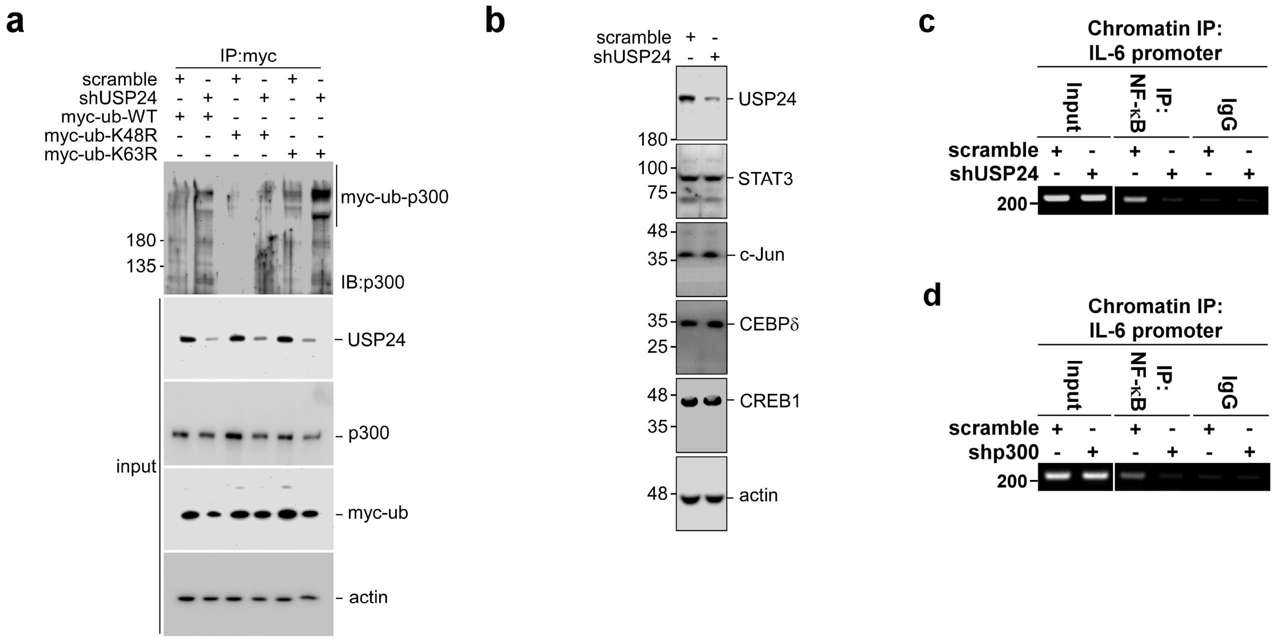

Supplementary Figure 3 - USP24 regulates IL-6 expression by stabilizing p300. a The plasmids, pCMV-Myc-Ubi-wt, pCMV-Myc-Ubi-K48R and pCMV-Myc-Ubi-K63R, were transfected into scramble- and USP24-knockdown A549 cells to study the ubiquitination of p300. **b** The lysates were harvested from scramble- and USP24-knockdown M2 macrophages for studying the indicated protein levels by Western blotting. **c** Recruitment of NF-κB to the promoter of IL-6 in scramble- and USP24-knockdown M2 macrophages were analyzed by chromatin immunoprecipitation assay. Anti- NF-κB antibody was used, and IL-6 promoter was analyzed by PCR. **d** Recruitment of NF-κB to the promoter of IL-6 in scramble- and p300-knockdown M2 macrophages was analyzed by chromatin immunoprecipitation assay. Anti- NF-κB antibody was used and IL-6 promoter was analyzed by PCR.

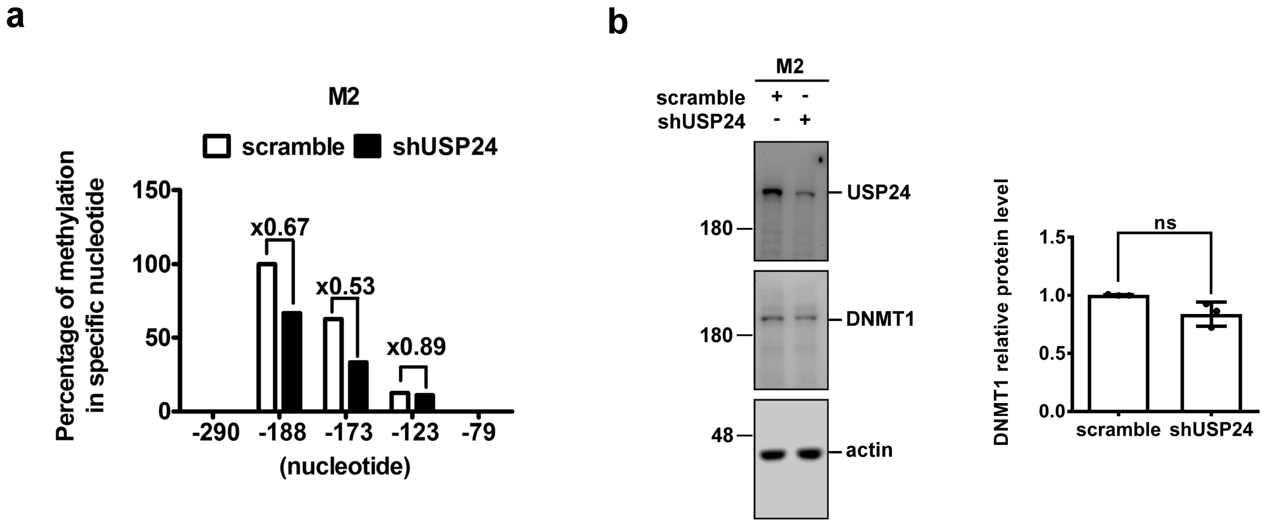

Supplementary Figure 4 –USP24 regulated IL-6 expression in M2 macrophages is DNMT1 independent. **a** Methylation sites in between -375 and -19 region was analyzed by using bisulfite sequencing. PCR products were inserted into yT&A vectors and amplified with competent cells. Sequences of the colonies derived from these competent cells were analyzed by Mission Biotech. Methylation status of each site analyzed by bisulfite sequencing in M2 macrophages was represented in percentage. **b** The lysates were harvested from USP24-knockdown M2 macrophages to study the indicated proteins levels with Western blotting (n = 3). Data are shown as mean ± SEM, two-tailed unpaired Student's t-test, ns for not significant, ns for not significant.

Supplementary Figure 5

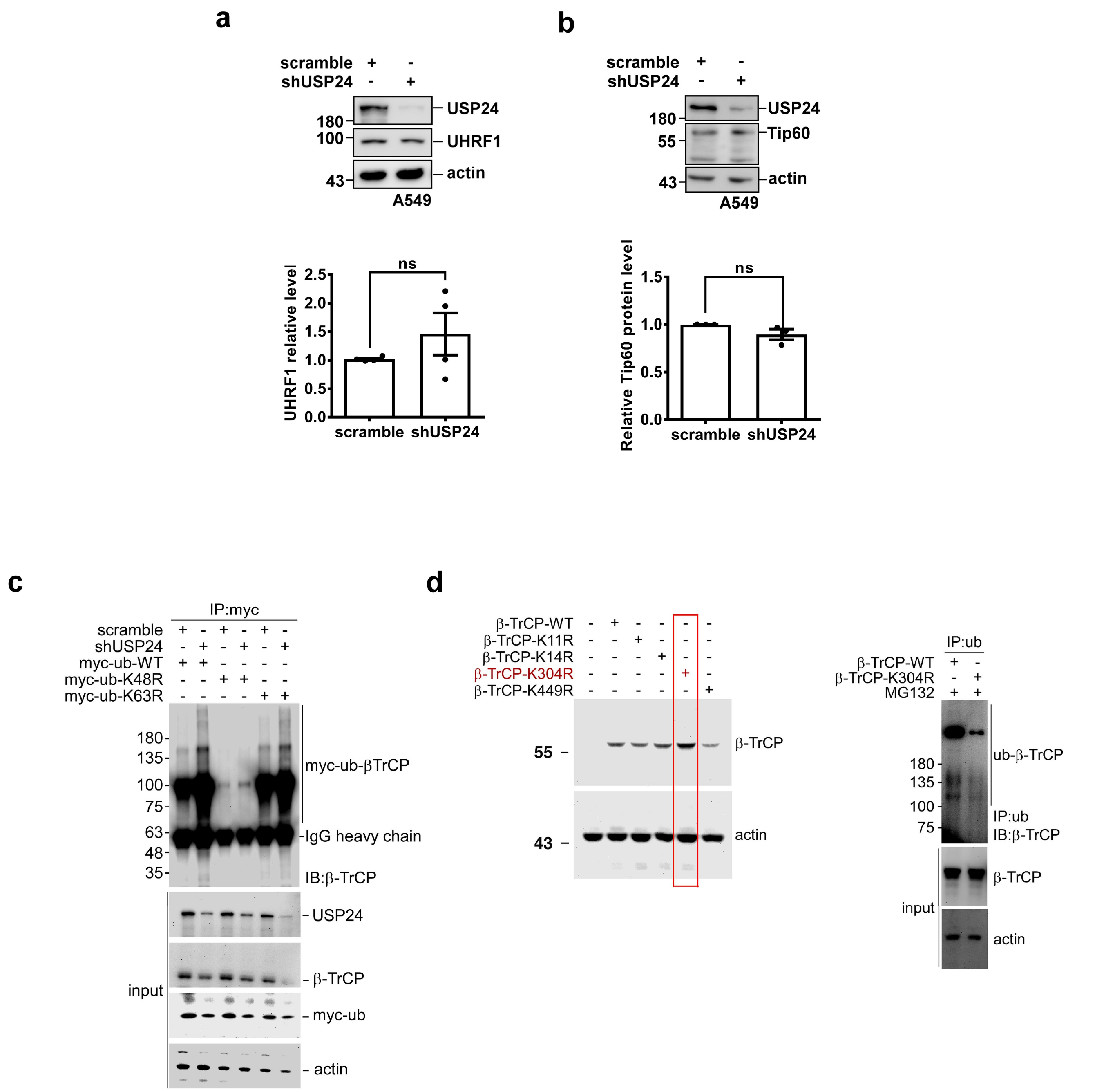

Supplementary Figure 5 – The level of DNMT1 regulatory proteins in USP24 knockdown cells. a UHRF1 level was examined in scramble- and USP24-knockdown A549 cells by Western blotting (n = 3). **b** Tip60 level was examined in scramble- and USP24-knockdown A549 cells by Western blotting (n = 3). **c** The plasmids, pCMV-Myc-Ubi-wt, pCMV-Myc-Ubi-K48R and pCMV-Myc-Ubi-K63R, were transfected into scramble- and USP24-knockdown A549 cells to study the ubiquitination of β-TrCP. **d** Several indicated lysine residues within β-TrCP were mutated individually and transfected into A549 cells to study the protein level (left panel) and ubiquitinated signal (right panel) by Western blotting. The potential ubiquitination sites responsible for β-TrCP protein stability was marked with red words and representative Western blotting result was indicated with red rectangle. Data are shown as mean ± SEM, two-tailed unpaired Student's t-test, ns for not significant, ns for not significant.

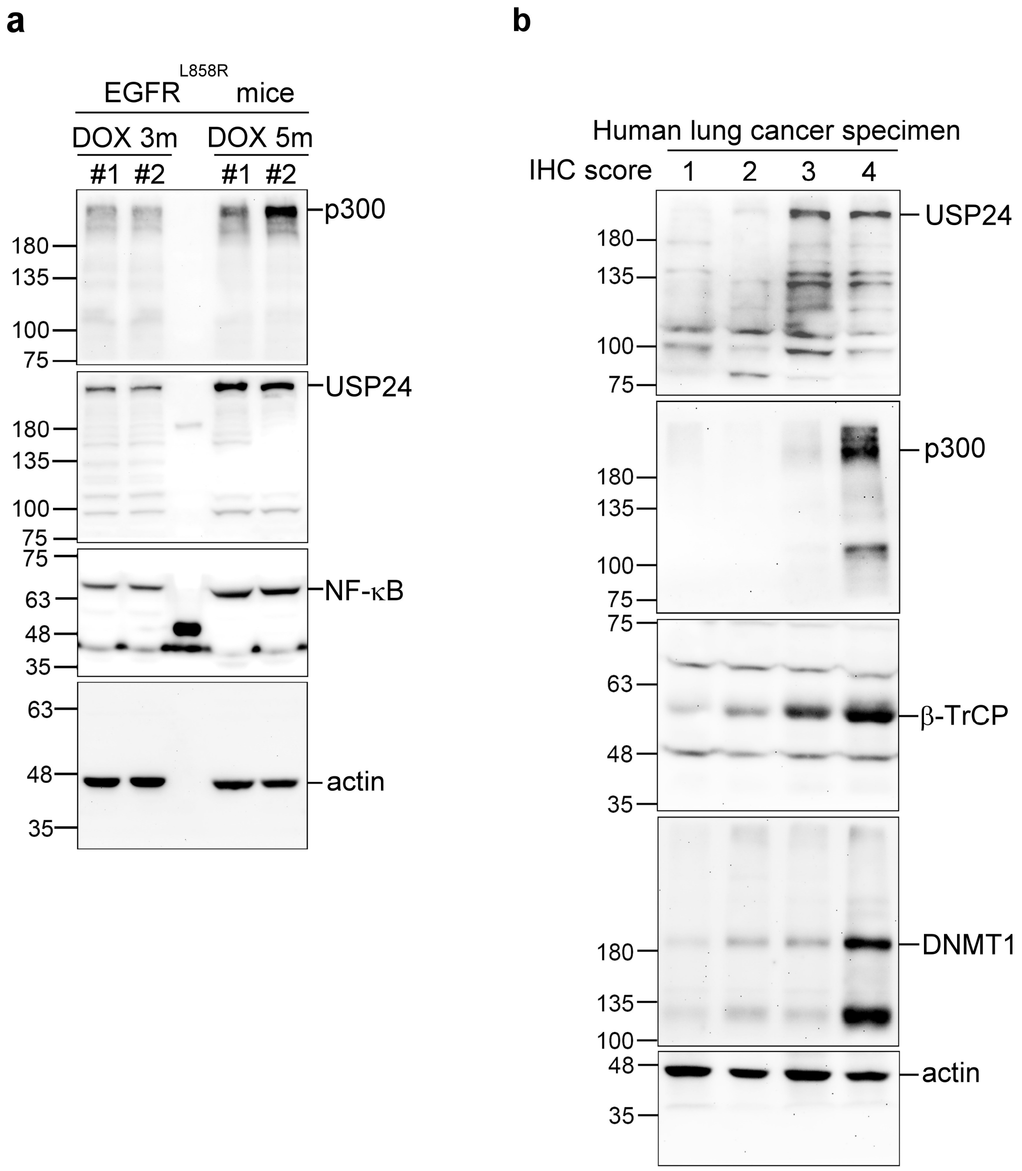

Supplementary Figure 6 – Validation of indicated antibodies by Western blot analysis. The specificities of the antibodies used in immunohistochemistry assays were validated by using Western blotting. **a** The same samples shown in Fig. 8a were analyzed by Western blotting with indicated antibodies to confirm the protein levels of p300, USP24, NF-κB. **b** The same samples shown in Fig. 8b to define the IHC scores were analyzed by Western blotting with indicated antibodies to validate the antibodies specificities.

Supplementary Table

Supplementary Table 1

Sequences of primers used for Q-PCR, Chip PCR, bisulfite sequence and single mutations

**RT-PCR and Q-PCR primers**		
**Gene**	Forward primer	Reverse primer
**USP24**	5′- CAGTTGTGCTCTCCTGTGGA-3′	5′- AGGGATTTCTCCTGCTCCAT-3′
**CD206**	5′-GAGCAGGTGGAAGATCTATG-3′	5′-CATAGTCAGTAGTGGTTCCG-3′
**GAPDH**	5′-CCATCACCATCTTCCAGGAG-3′	5′-CCTGCTTCACCACCTTCTTG-3′
**IL-6**	5′-AGAGTAACATGTGTGAAAGCAG-3′	5′-TCAGGACTTTTGTACTCATCTG-3′
**IL-8**	5′-AGACATACTCCAAACCTTTCCA-3′	5′-TTATGAATTCTCAGCCCTCTTC-3′
**IL-10**	5′-TTGTCTGAGATGATCCAGTTT-3′	5′-ACTCACTCATGGCTTTGTAGA-3
**NF-κB**	5′-CTATGAGGATGATGAGAATGG-3′	5′-ACCAGAGGGTAATAGGTGAAC-3′
**CCL2**	5′-CCTCGTGTTATAACTTCACCA-3′	5′-GTCTTGAAGATCACAGCTTCTT-3′
**CCL17**	5′-TTCCCCTTAGAAAGCTGAAG-3′	5′-CTTCACTCTCTTGTTGTTGG-3′
**CCL22**	5′-GTGGTGAAACACTTCTACTGG-3′	5′-TGGCTCAGCTTATTGAGAAT-3′
**CCL24**	5′-GACCATAGTAACCAGCCTTCT-3′	5′-TTCTCTTGGAAACAAAGAACA-3′
**ChIP PCR primers**		
**IL-6 promoter**	5′-AAATGCCCAACAGAGGTCAC-3′	5′-TTCCCTCAGGATGGTGTCTC-3′
**IL-6 promoter (putative NF-κB binding region)**	5′-TGCACTTTTCCCCCTAGTTG-3′	5′- GCCTCAGACATCTCCAGTCC -3′
**NF-κB promoter**	5′-GGGGTGGGGAAGTAATAGGA-3′	5′-GCGTTTCTCCTTAGCAGGTG-3′
**Bisulfite sequencing primers**		
**IL-6 promoter**	5′-GTTTGTTAATTTGGTTATTGAAAAA-3′	5′-CTCATAAAAAAATCCCACATTTAAT-3′
**β-TrCP Single mutation primers**		
**K11R**	5′-CGGTGCTGCAAGAGAGGGCACTCAAGTTTAT-3′	5′-ATAAACTTGAGTGCCCTCTCTTGCAGCACCG-3′
**K14R**	5′-GCAAGAGAAGGCACTCAGGTTTATGTGCTCTATGC-3′	5′-GCATAGAGCACATAAACCTGAGTGCCTTCTCTTGC-3′
**K304R**	5′-GCCGAAGTGAAACAAGCAGAGGAGTTTACTGTTTACAG-3′	5′-CTGTAAACAGTAAACTCCTCTGCTTGTTTCACTTCGGC-3′
**K315R**	5′-TTACAGTATGATGATCAGAGAATAGTAAGCGGCCTTCGA-3′	5′-TCGAAGGCCGCTTACTATTCTCTGATCATCATACTGTAA-3′
**K449R**	5′-TCTGGGGATAGAACTATAAGGGTATGGAACACAAGTACT-3′	5′-AGTACTTGTGTTCCATACCCTTATAGTTCTATCCCCAGA-3′
